# Systematic Review and Critical Analysis of Longitudinal Studies Assessing Effect of E-Cigarettes on Cigarette Initiation among Adolescent Never-Smokers

**DOI:** 10.3390/ijerph20206936

**Published:** 2023-10-18

**Authors:** Bertrand Dautzenberg, Stéphane Legleye, Michel Underner, Philippe Arvers, Bhavish Pothegadoo, Abdelhalim Bensaidi

**Affiliations:** 1 Sorbonne Université & ex CHU Pitié-Salpêtrière (APHP), 14 Avenue Bosquet, 75007 Paris, France; 2 Institut Arthur Vernes, Tabacologie, 75006 Paris, France; 3 Ensai & Cesp, 35172 Bruz, France; stephane.legleye@inserm.fr; 4 Université Paris-Saclay, Faculté de Médecine UVSQ, 91190 Gif-sur-Yvette, France; 5 Centre Hospitalier Laborit, Unité de Recherche Clinique Pierre Deniker, Tabacologie, 86000 Poitiers, France; mike.underner@orange.fr; 6 7ème Centre Médical des Armées, Quartier De Reyniès, D1075, Consultation Addictologie et Tabacologie, 38760 Varces-Allières-et-Risset, France; philippe.arvers@gmail.com; 7 Hôpital Maison Lafitte, Unité de Cardiologie, 78600 Maison Lafitte, France; bhavish.pothegadoo@gmail.com; 8 Hôpital de Nanterre, Diabétologie, 92000 Nanterre, France; abdelhalimbensaidi@gmail.com

**Keywords:** systematic review, adolescents, cigarette, e-cigarette, gateway effect, diversion effect, longitudinal study, selection bias

## Abstract

Prospective longitudinal studies mainly conclude on a causal role of e-cigarettes in the initiation of cigarettes in flagrant contradiction with conclusions drawn from epidemiology and other studies showing a sharp decline in cigarette use in parallel with the spread of e-cigarette use. This systematic review explores the reasons for this discrepancy. Methods. Among 84 publications on e-cigarette/cigarette association in adolescents identified in the Medline database from 2011 to 2022, 23 concern 22 never-smoker longitudinal sub-cohorts. Results. A link between e-cigarette experimentation at T1 and cigarette initiation at T2 is reported in sub-cohort analyses of never-smokers (AOR: 1.41 to 8.30). However, studies exclude 64.3% of T1 e-cigarette experimenters (because of dual-use) and 74.1% of T2 cigarette experimenters. With this study design, e-cigarettes contribute only to 5.3% of T2 cigarette experimentation, casting major doubt on the external validity of results and authors’ conclusions that e-cigarettes have a significant effect on the initiation of cigarettes (*Gateway effect*) at the population level. This sub-cohort design prohibits highlighting any *Diversion effect*, which is the most likely mechanism accounting for the competition between these two products. Conclusions. While nicotine abstinence remains the best medical option, over-regulation of e-cigarettes because of misinterpretation of longitudinal study results may be detrimental to public health and tobacco control.

## 1. Introduction

Getting adolescents hooked on nicotine and tobacco has been the goal of the tobacco industry for ¾ of a century [1]. The earlier the initiation to tobacco, the more intense the tobacco dependence [2], the greater the difficulty in quitting, and the greater the long-term profit prospects for long-term profits for the tobacco industry. Protecting adolescents from initiation and consumption is, therefore, legitimate and effective in terms of public health. Most developed countries have set themselves the objective of quitting smoking.

The place of the e-cigarette in this approach is debated in the literature [3,4,5,6,7,8]. The e-cigarette, also called ENDS, the vape, or the electronic cigarette, became available between 2010 and 2013, according to countries. Some argued for making e-cigarettes a medicine, others wanted to make them a tobacco product, others wanted to make them a consumer good, and others wanted to ban them [9,10]. In 2014, the European Parliament decided in its 2014/40/EU directive that the e-cigarette was an *everyday consumer product* associated with tobacco that requires a precise framework.

Among adults, the replacement of tobacco cigarettes by e-cigarettes raises little concern among public health professionals because they are much less dangerous, they are consumed almost exclusively by smokers and former smokers, and they are well-evaluated products for smoking cessation [11,12]. In the 2023 Cochrane meta-analysis [13], e-cigarettes are reported as safe and have the highest AOR versus placebo for smoking cessation (AOR = 2.37 (1.43–3.24)), when the AOR is 2.33 (2.02–2.68) for varenicline and 1.93 (1.61–2.34) for nicotine patch + oral nicotine replacement therapy. In scientific incoherence with these results, in many countries, e-cigarettes are not positively recommended by scientific organizations or health authorities for smoking cessation; it is sometimes an open choice for the patient. In some countries, e-cigarettes are discouraged or prohibited when tobacco remains authorized. One of the arguments for prohibiting e-cigarettes in these countries is the allegation of the risk of e-cigarette experimentation on the initiation of cigarette use.

Regarding adolescents, the role of e-cigarettes has been questioned since 2013: will their emergence increase, stabilize, or reduce the rate of regular smokers? [14,15,16,17,18,19].

Most prospective longitudinal studies on non-smokers conclude that e-cigarette initiation favors cigarette initiation (*Gateway effect*). This observation was reinforced by the publication of meta-analyses that conclude in a similar way [20,21,22,23,24,25]. This is more convincing in that there are many pertinent mechanisms to back it up. One is that e-cigarette use normalizes tobacco use and makes it more likely that people will try combustible tobacco cigarettes. Another is that e-cigarette use could lead to nicotine addiction, which can increase the risk of using tobacco products. Additionally, e-cigarette use may expose users to harmful chemicals and could increase their risk of developing nicotine dependence. Furthermore, the historic struggle to regulate smoking and its recognition as a major cause of harm to public health has prompted caution about the tobacco industry’s innovations, leading to suspicion about e-cigarette marketing. Nevertheless, behind the coherent and intellectually reassuring character of this body of evidence useful to public health, it appears that the claims of causality of these longitudinal studies are in flagrant contradiction with the continuing decline in smoking and the concomitant rise of e-cigarette use in the adolescent population.

The goal of this paper is to investigate this paradox: how can scientists argue that e-cigarettes contribute to the spread of smoking under these conditions, even though they base their judgment on the best instrument available for studying causality, namely longitudinal surveys?

The initial analysis plan for this review included an analysis of inconsistencies between different types of studies and an in-depth examination of risk factors and confounders related to e-cigarettes and cigarettes. But, after having tabulated the data from the longitudinal studies, the drastic selection before the inclusion of the sub-cohorts appeared to us to be the most probable source of bias, far ahead of the analysis of the factors initially anticipated. Consequently, we moved our analysis to focus on the sub-cohorts of non-smoking adolescents who had been assessed with a unilateral design for the role of e-cigarettes in cigarette initiation in never-smokers during follow-up. We also conducted an internal consistency analysis of the raw data on the published sub-cohorts and the authors’ conclusions. The analysis of non-longitudinal studies in this review focuses on studies that assess the bilateral effects (*Diversion effect* and *Gateway effect*) on trajectories, pseudo-cohorts, and the first use of e-cigarettes or cigarettes.

## 2. Materials and Methods

### 2.1. Protocol

The initial protocol of analysis was registered in the PROSPERO database on the number CRD42023396960 on 15 February 2023, and the PRISMA checklist is in the report in Supplementary Material Table S1.

### 2.2. Identification of Studies in Analysis

**Figure 1:**
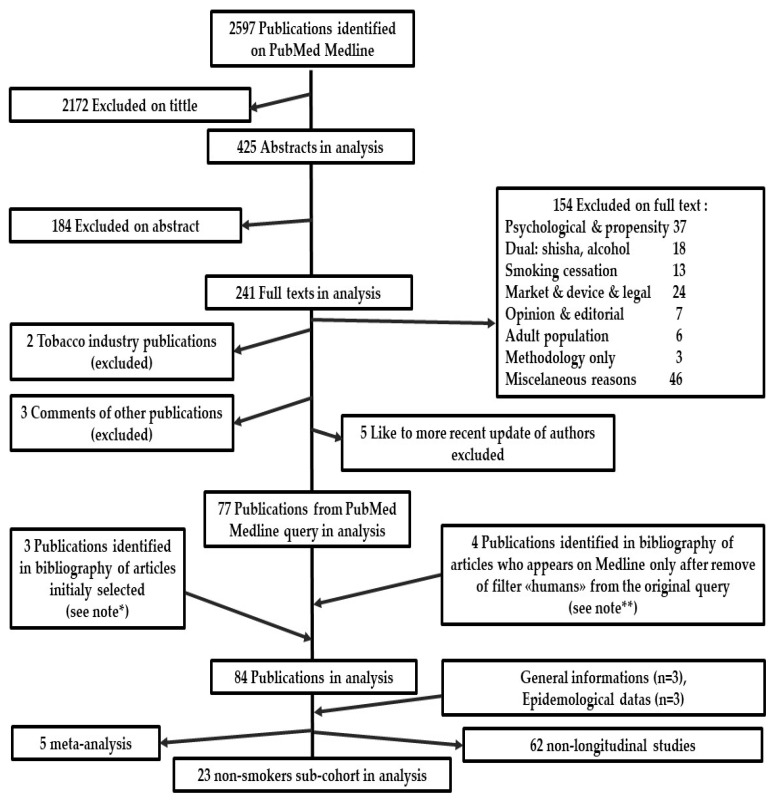
Flowchart of publication selection. (note: “**” concern 4 publications identified only after removing of filter “humans” from the original query [20,22,26,27]); “*” concern 3 publications identified in bibliography of selected articles who has been added [28,29,30].

Medline search was conducted between 2011 and March 2023, applied using the “Human” filter and three keywords (e-cigarette, cigarette, and adolescent) and their synonyms (Supplementary Material Table S2). The article selection flowchart is presented in Figure 1. 

### 2.3. Article Inclusion

Each initially retrieved article was analyzed by two authors and ranked either as “accepted”, “to be discussed”, or “rejected”. Articles classified as “to be discussed” by both authors or with conflicting judgments, were analyzed a second time. In the absence of consensus, the judgment of a third author was requested to obtain a definitive inclusion in the present review. For articles with internal inconsistencies of figures, in absence of online correction, priority was given to the abstract, then to the result section, then to the tables or figures, then to the discussion, and finally to the online Supplementary Material.

### 2.4. Longitudinal Studies

#### 2.4.1. Presentation of Sub-Cohort Results

For the presentation of longitudinal studies, the presentation of sub-cohort is briefly tabulated with key cohort characteristics, adjusted odds ratios (AORs with confidential interval 95% CI), and row figures. The data are presented at inclusion (referred to as T1) and at the end of follow-up (referred to as T2).

We identified 6 meta-analyses that included selected-sub-cohorts [20,21,22,23,24,25]. The five more recent were considered in parallel to the individual sub-cohort studies.

#### 2.4.2. Reinstating of Smokers Excluded in the Sub-Cohorts to Reconstitute Cohorts

We reconstituted the original cohorts by reinstating “cohort” tobacco smokers excluded before T1 to obtain the overall number of smoking initiations and assess the number of dual-users (e-cigarettes + cigarettes) excluded at T1 (when data are available).

In this systematic review, we will use the term “sub-cohort” to indicate the fraction of never-smoking adolescents included in the initial cohorts and who are named “cohort” in the original articles. We reserve the term “cohort” for the entire cohort, including smokers, described in the article or reconstituted by reintegration of never-smokers.

#### 2.4.3. Analysis of the Coherence between Results and Conclusions of Longitudinal Studies

To assess the coherence between results and conclusions of longitudinal studies, two scores ranging from 0 to 3 were constructed as follows:

The causality score assesses the general causal role of e-cigarettes on cigarette initiation attributed by authors in conclusions (extrapolated from data from their sub-cohorts excluding exclusive smokers and dual-users at T1).

The score of requests to change the e-cigarette regulation (based on the results of their sub-cohorts who assess only *Gateway effect* and not *Diversion effect*).

A combined score was established by adding the two scores.

The methodology used is detailed in Supplementary Material Table S3.

### 2.5. Other Studies

With the new plan of analysis, we focus only on epidemiology, modeling studies, and studies on age and chronology of initiation.

#### 2.5.1. Epidemiology

We limited the epidemiological research to the USA, where most studies were conducted, and to France, with particular attention to select studies representative of 17–18-year-old teenagers.

#### 2.5.2. Modeling Studies

Modeling analyses using longitudinal epidemiological data were carried out to test certain hypotheses relating to the causal effect of e-cigarettes in the transition to smoking, and the reverse was reviewed [19,30,31].

#### 2.5.3. Studies on Age and Chronology of Initiation

We analyze studies reporting the age and the chronology of initiation in the whole population.

#### 2.5.4. Role of Flavors and Nicotine Concentration

The role of the e-cigarette/cigarette interaction of aromas and nicotine in the e-liquids on the experimentation and use of e-cigarettes is identified and analyzed in the studies that mention them.

## 3. Results

### 3.1. Transition from E-Cigarette Use to Cigarette Initiation among Never-Smokers in the 22 Longitudinal Sub-Cohorts

All the 22 sub-cohorts started their follow-up between 2012 and 2019 (Table 1). 

One article used the same data to conduct and publish two different specific analyses [35,41]. Among the 23 publications, 18 considered e-cigarette experimentation at T1 as an inclusion event. At T2, publications considered cigarette experimentation (17/23), use (8/23), and daily use of cigarettes (3/23) at the end of follow-up [37,49,51]. Most of the sub-cohorts (12/23) report only the effect of experimentation at inclusion and at the outcome (at minimum, one or two e-cigarette puffs at T1 and one or two cigarette puffs before T2). Daily e-cigarette use at T1 is not analyzed by authors (0/23) (Supplementary Material Table S4).

The risk of cigarette smoking initiation at T2 in the 22 sub-cohorts is reported in the 23 publications as adjusted odds ratios (AORs) (*n* = 20) or as relative risk RR (*n* = 3) [38,39,46]. The AOR is adjusted for, on average, 6.64 confounding factors. The AOR is, on average, 50.7% of the crude OR reported in the studies. The main results are presented in Table 2.

### 3.2. Meta-Analysis

The five most recent meta-analyses that included selected sub-cohorts are presented in Table 3. The meta-analysis by Chatterjee et al. [20] does not include the most recent studies. In the five meta-analyses, e-cigarette experimentation at T1 increases the risk of smoking experimentation at T2 (AOR range from 2.93 [23] to 4.06 [24]). These AORs vary according to the heterogeneity of the studies and many factors such as the sample size, the year of the study, the duration of follow-up (>12 months reduces the OR from 11.32 to 3.54), and the continent. The meta-analysis publications do not highlight the selection biases of the sub-cohorts. The inclusion of adjustment factors, especially the propensity to smoke, reduces the odds ratios and sometimes the confidence intervals.

### 3.3. Reinstating of Excluded Smokers in the 22 Sub-Cohorts Analyzed to Reconstitute Original Cohorts

**Figure 2:**
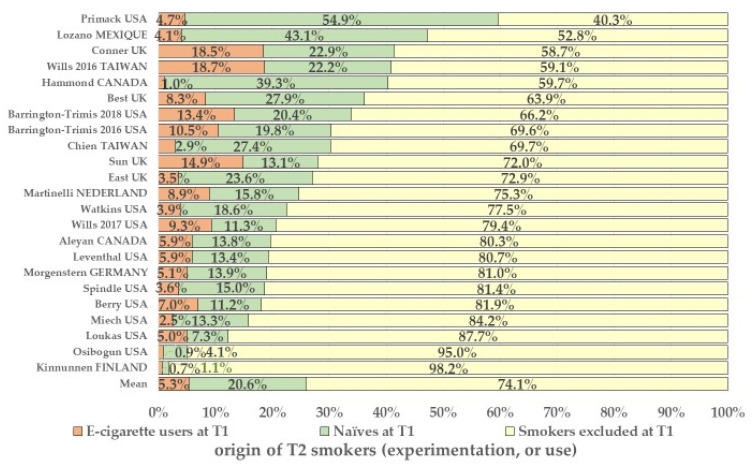
Origin of smokers at T2 (%) after reconstitution of the 22 cohorts by reinstating of smokers in the sub-cohorts (23 publications). Note: mean is the ratio (%) of the number of adolescents in each of the three categories who had experimented with cigarettes at T2/the total number of subjects in the original cohorts.

The number of excluded smokers at T1 is reported as a figure or percentage by the authors of 18 among the 22 studies or obtained as reported in Supplementary Material Table S5 in the 5 other studies. The distribution of T2 cigarette smokers is reported in Figure 2.

**Figure 3:**
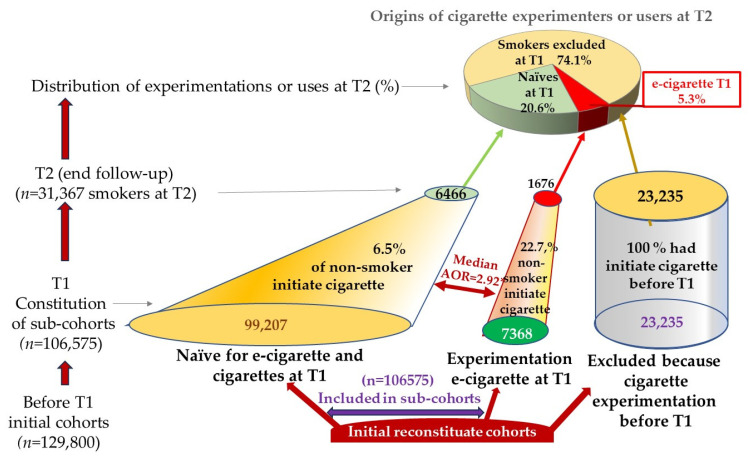
Estimation of the distribution of smokers (at least experimentation) at T2 after reinstating in the sub-cohort smokers excluded at T1.

In all, 23,225 smokers at T1 were excluded from the initial cohorts to constitute the sub-cohorts that comprised 106,575 non-smokers effectively included and analyzed at T1, that is, 17.9% of the 129,800 adolescents in the original population cohorts (Figure 3 and Supplementary Material Table S6). In the original cohorts, 74.0% of T2 smokers had thus experimented with cigarettes before their exclusion in sub-cohort at T1; 20,6% had never used e-cigarettes; and only 5.3% (1671) had a link (causal or not) with e-cigarette use at T1.

**Figure 4:**
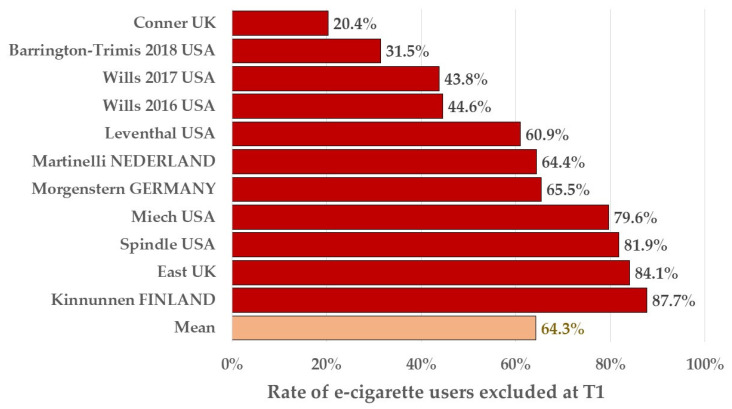
Rate of e-cigarette users excluded at T1 because of dual-use of e-cigarettes/cigarettes. Note: Mean is calculated as the ratio of the total number of adolescents excluded because of dual-use reported to the total number of adolescents in the initial cohort.

Because of the exclusion of all adolescents who had experimented with cigarettes (only cigarette or dual-use of cigarette + e-cigarette) at T1, almost two-thirds of the e-cigarette experimenters (64.3%) were excluded in the sub-cohort analysis according to the results of the 11 publications where data on dual-use were available at inclusion, introducing a severe bias (Figure 4).

### 3.4. Causality and Recommendation to Change the Regulation

The hypothesis or assertion of a cause-and-effect relationship between e-cigarette initiation on cigarette experimentation and use at follow-up appears in the abstracts, discussions, or conclusions in 22/23 publications; recommendations to limit the development of e-cigarettes through regulatory measures appear in 16/23 publications (Table 4) and was detailed in Supplementary material Table S7. Three of the four studies with the maximum combined score (6) are from North America, 1/4 are from Asia, and 0 are from Europe. The only study with a value of zero for the combined score is from Europe.

**Figure 5:**
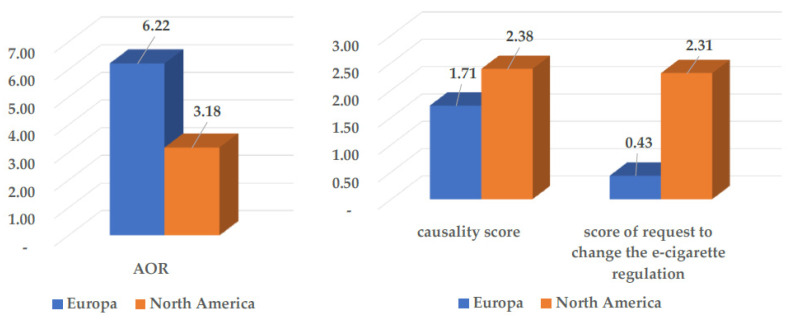
Comparison of the combined AORs as reported in meta-analysis of O’Brien [24] and causality scoring of authors’ conclusions and scoring of requests to change the rules in the 15 North American studies and the 7 European studies.

While O’Brien’s meta-analysis [24] reports a mean combined odds ratio (ORs) two times lower in North American studies than in European studies (ORs = 3.18 vs. ORs = 6.22) for initiating cigarettes after experimenting with e-cigarettes, the conclusions of the authors are two times higher in denouncing a causality effect and making a much stronger demand for legislative changes in North America (mean combined score 4.5/6) than in Europe (mean combined score 2.1/6) (Figure 5).

Wills et al. published three analyses [35,41,54] of the same longitudinal cohort data. Considering the whole cohort without the exclusion of smokers at T1 and with an auto-regression model [54], they conclude the absence of a causal link between initial experimentation of e-cigarettes and cigarette consumption at T2. The authors explain that e-cigarette experimentation is only a marker of personal and environmental factors. Considering only a sub-cohort obtained with the same design as the 21 other sub-cohorts, the same authors provide opposite conclusions with the *Gateway effect* (combined score 5/6 in the present analysis) [35,41].

Indeed, the *Gateway effect* appears only in the selected sub-cohorts but not at the population level in the Wills et al. studies [35,41,54], which analyze the same group of adolescents.

When the whole original cohort is considered, the *Dispersion effect* is dominant and totally masks a possible *Gateway effect.*

The *Gateway effect* is identified only in sub-cohort after exclusions of initial smokers.

The studies of Wills et al. [35,41,54] question the validity at the population level of the sub-cohort analyses.

### 3.5. Reverse Analysis of the Influence of Cigarette Use at T1 on E-Cigarette Use at T2

Among the 22 prospective sub-cohorts, 3 also include an inverse relationship between the effect of cigarette smoking at T1 on initiation to e-cigarettes at T2 [34,44,52] in tobacco and e-cigarette naïve adolescents. In addition, five other studies [27,42,55,56,57] (mainly based on pseudo-cohorts) report that the average AOR for experimentation is similar for the two pathways: the average AOR = 4.62 for the link between e-cigarette initiation at T1 and cigarette initiation at T2 and is 5.37 for the inverse pathway. However, the median AOR for the association between regular use of e-cigarettes at T1 and regular use of cigarettes at T2 is 4.18, while the median AOR for the inverse pathway is 7.8 (Supplementary Material Table S8).

### 3.6. Other Studies

#### 3.6.1. Cigarette Smoking in Adolescents after 11 Years of Vaping (2011–2022)

In the United States, e-cigarettes became available to adolescents in 2009–2010 [27] but were not widely available and used until 2013–2014 [26]. Epidemiological data before 2009–2010 showed a steady decline in the proportion of cigarette smokers in New Zealand [58], the United States [15,27,29,37,47,59,60,61], Canada [58], France [16,62,63], the United Kingdom [30], Mexico [48], and Finland [45], as well as in most developed countries where data exist.

**Figure 6:**
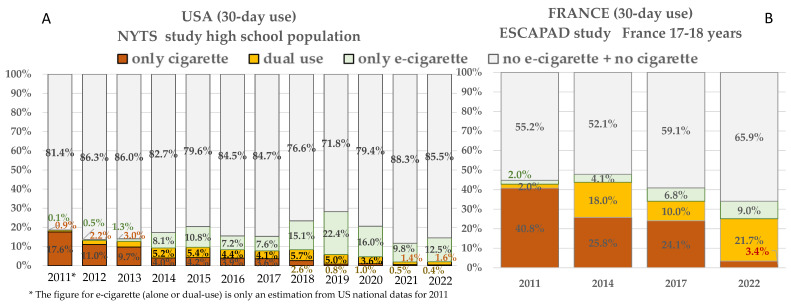
Recent evolution of 30-day consumption of cigarettes or e-cigarettes at 17–18 years according to (**A**) NYTS USA [16] and (**B**) ESCAPAD France [15].

The most recent data on consumption in the USA and France are NYTS [15] and ESCAPAD studies [16]. In the USA, the proportion of cigarette users (only cigarettes in the last 30 days or dual-users) decreased from 17.7% in 2011 to 2.0% in 2022 [15] (Figure 6A). In France, the proportion of cigarette users (with or without e-cigarette use) in the past 30 days decreased from 42.8% to 25.1% between 2011 and 2022 [16] (Figure 6B), while the proportion of daily smokers decreased from 31.5% to 15.6%. The proportion of naïve adolescents (no tobacco cigarette and no e-cigarette) was rather stable in the USA, ranging between 81.4% (2011) and 85.5% (2022) [16] but increased from 55.2% to 65.9% in France from 2011 to 2022 [15].

#### 3.6.2. Escalation to Regular Use According to the First Product Experimented

Near ¾ of the population studied concerned only the experimentation of cigarettes or e-cigarettes.

Some studies [64,65,66,67,68,69] analyzed escalation and regular use of the product.

In five studies, the risk of regular cigarette smoking is nearly four times higher with a first initiation with cigarettes than with e-cigarettes. In three studies, the OR of becoming a regular smoker is almost three times higher after a first initiation with cigarettes than with e-cigarettes. The association with regular use is eight times higher for regular cigarette use after a first initiation with cigarettes than with e-cigarettes.

Shahab et al. [70] report that after the first use of an e-cigarette instead of the first use of a cigarette, the risk of smoking in the last 30 days is lowered, with an AOR = 0.48 (0.36–0.62), just like that of an ordinary cigarette user, with an AOR = 0.26 (0.19–0.35).

The transition from experimentation to daily use is found in the study by Legleye et al. [62]: nearly half of the 24,111 adolescents aged 17–18.5 who had tried cigarettes became daily cigarette smokers (46.3%), compared to 18.7% of those who had tried electronic cigarettes first. They report that “*experimenting with e-cigarettes first (as opposed to tobacco first) appears to be associated with a reduced risk of daily smoking*”, with an RR = 0.58 (0.54–0.62).

#### 3.6.3. Modelling of the Evolution of Cigarette Consumption in Adolescents 

Selya et al. [19] report, by modeling NYTS study data, that a substantial *Diversion effect* of cigarette use replaced by e-cigarette use is necessary to explain the observed trend in nicotine use among American youth and that this diversionary effect must outweigh any contrary catalytic effect (*Gateway effect*). Hallingberg et al. [31] found an increased decline in smoking in the UK since 2010 (OR = 0.88 (0.86–0.90)). A New Zealand study [30] concludes that the *Diversion effect* is the most plausible in their mixed model, explaining 54% of the reduction in cigarette consumption in the absence of the *Gateway effect*.

#### 3.6.4. Age and Chronology of E-Cigarette and/or Cigarette Initiations

The mean age at the cigarette experimentation is below that of the first e-cigarette [71,72]. Foxon et al. [59] reported that the age at cigarette smoking initiation among American adolescents, which was stable at 11.7 years between 1990 and 2013, has risen sharply since, by +0.7 years per year between 2013 and 2018, with the advent of e-cigarettes.

Evans-Polce et al. [73] note that nearly half (47.4%) of those who experiment with e-cigarettes do not switch to cigarettes among 9858 adolescents who were initially lifetime e-cigarette or cigarette users. In 27.1% of cases, the first product is the e-cigarette among the dual-experimenters. The cigarette is 2.9 more often experimented before the e-cigarette (18.5%) than the reverse (6.9%). Among consumers of one or the other product, the first initiated product is the cigarette for 72.9% of the cases. For this vast majority of adolescents, the e-cigarette cannot have an important *Gateway effect* on cigarette initiation.

#### 3.6.5. Role of Flavor 

Several studies in adolescents or young adults suggest that [74,75] flavors promote the uptake of e-cigarette e-cigarettes because they appear less hazardous and more appealing, especially when there are numerous flavors to choose from [76,77]. The authors conclude that this may increase nicotine dependence and could favor the transition to tobacco use.

#### 3.6.6. Role of Nicotine

The role of nicotine is poorly assessed in the longitudinal and transversal studies reviewed [78]. The rate of use of nicotine-free e-cigarettes is significant but not properly reported. The huge differences in national regulations of e-liquid nicotine contents may contribute to the variability across countries, which calls for future research. However, it does not change the overall picture, as we considered e-cigarettes and e-liquids (with nicotine and or flavors), that is, vaping, as a primary variable of interest.

## 4. Discussion

### 4.1. Identification of the Cause of the Discrepancy between Longitudinal Studies and the Others

In preparing this general review, we anticipated the need for a detailed analysis of the personal and environmental factors contributing to the initiation and use of cigarettes and e-cigarettes among adolescents [79,80,81,82,83] to understand the discrepancy between the conclusions of longitudinal studies and those of other studies possibly leading to decisions unfavorable to public health [84]. During the analysis, it quickly became apparent that this discrepancy was mainly due to a misinterpretation of the results of the sub-cohorts that were used to draw conclusions at the population level. We thus focused on the internal inconsistencies between the results and the conclusions in the 22 published longitudinal sub-cohorts assessing cigarette/e-cigarette association.

The studies on experimentation or use of tobacco products other than cigarettes, smoking tobacco, non-smoking tobacco, or cannabis studies are heterogeneous. The complex bi-directional links between these consumptions and those of cigarettes and e-cigarettes were identified and only poorly quantified and not reported in our review [85,86,87].

### 4.2. Longitudinal Studies of Never-Smokers Are Not Representative of Whole Cohort of Adolescents

Data collected in sub-cohort reporting a *Gateway effect* concern only 5.3% of all cigarette initiations at T2 and 1.3% of the population of adolescents of the original cohort. The exclusion of all early initiators of cigarettes, and many early initiators of e-cigarettes, because they were dual-users at T1, without information on the timing of initiation of the two products, has two consequences: (1) it reduces the external validity of the analyses and conclusions, causing them to only be valid in a small subsample of adolescents and cannot be extrapolated to the initial cohort and (2) it prevents analyzing the potential protective *Diversion effect* of e-cigarettes on cigarette consumption.

### 4.3. The Unilateral Design of Longitudinal Study Rules out Any Evidence of a Diversion Effect

The studies on the chronology of initiation report that the e-cigarette is more often initiated after the cigarette, excluding that the e-cigarette may cause the initiation of the cigarette in this situation [62,63,64,65,66,67,68,69,70]. Conversely, most studies that analyze escalation report that after the initiation of cigarettes in this situation, the rate of cigarette use in the month is much lower than after initiation by cigarette first [64,65,66,67].

The e-cigarette thus has a *Diversion effect* on the transition from experimentation to regular cigarette use. Choosing the naïve individuals as a reference group at inclusion instead of the cigarette experimenters practically leads to high-risk ratios for the transition to regular tobacco smoking. This is because experimenting with e-cigarettes at a given age is a sign that someone is more likely to take risks.

The *Diversion effect* of e-cigarettes is not well documented for smoking cessation in adolescents. Wang et al. [88] show that students under 18 declare that the first reason to use e-cigarettes is the intention to reduce or quit smoking. However, studies in adolescents do not confirm an increase in quitting, which is only observed in young adults [88,89,90], but the exploration of tobacco cessation is complex in adolescents who are not established smokers and need further studies.

### 4.4. The Unilateral Design of Longitudinal Study Rules out Any Evidence of a Diversion Effect

The selection made in the sub-cohorts comes at a point in the product diffusion cycle during adolescents’ lives: having experimented with or smoked a product before a certain age provides information about adolescents’ appetite for risk. It is predictive of more frequent and varied experimentation later on. If the selection is made before the end of adolescence and, therefore, before the end of the experimentation calendar, not all exposures are taken into account, and only part of young people’s consumption trajectories are analyzed. This introduces a potentially significant bias in the estimation of the causal effect. Thus, selection based on age at inclusion induces selection on the basis of the propensity to consume psychoactive substances. Considering age at exposure to tobacco and e-cigarettes is fundamental to understanding the causal effect of these products at a given point in time, and only a comprehensive analysis of all events during adolescence will enable an unbiased estimate of an average causal effect over a generation. The 5.3% of smokers at T2 compared to the expected 1.7% having vaped at T1 in the reconstituted cohort may as well be evidence of a selection of a higher risk group than of a causal effect.

### 4.5. Reasons for the Error in Design and Interpretation of Adolescent Longitudinal Studies

Our analysis points to a methodological problem in the design of the longitudinal studies. However, it does not explain why hundreds of authors, dozens of reviewers, publishers, and funding organizations involved in the process did not clearly point out the discrepancy between the results based on sub-cohorts and the authors’ general health conclusions. Some hypotheses may be raised: longitudinal studies are credited with a higher level of scientific evidence than cross-sectional studies and inspire great confidence in the authors, who may forget some limitations of the study designs.

The authors’ conclusions and the opposite trends in e-cigarette and tobacco use levels might have seemed compatible 10 years ago when smoking levels were still high, and e-cigarettes were just beginning to spread, but not in 2023.

Smoking tobacco is so harmful that the authors’ recommendations may have seemed entirely legitimate and natural. These factors may have contributed to reducing the attention paid to the details of the analysis methodology.

### 4.6. Consequence of the Abusive Non-Scientific Misinterpretation of Study by Authors of Longitudinal Studies

The misinterpretation of longitudinal studies influenced medical and scientific organizations, health authorities, and regulations on the protection of adolescents, which was one of the main arguments used to justify negative advertising and the e-cigarette ban.

The WHO 2023 report about tobacco in the world is a prototype of misinterpretation of sub-cohort results and disseminate recommendations not based of good sciences at population level and not based on epidemiology. The WHO states [17] that “ENDS (e-cigarette) are addictive and harmful, particularly for young people ENDS contain nicotine—the highly addictive substance in to-bacco. Using ENDS poses the risk of nicotine addiction, including among children and adolescents. Research findings show that non-smoking young people who use ENDS are more likely to become cigarette smokers, exposing them to the harmful effects of smoking, including addiction to tobacco”. The WHO report provides the only reference of a manifest of an NGO, Stopping Tobacco Organizations and Products (*STOP*), an organization financed by Blomberg Philanthropies, a very active anti-e-cigarette organization. This statement from the WHO without scientific reference is mainly based on the conclusions of the sub-cohorts analyzed in our systematic review.

### 4.7. Limitations of the Study

An important limitation of our systematic review is linked to the change in the analysis plan. The modifications of the initial analysis plan followed the first tabulation of the data after the selection of publications, which led us to discover a major bias, an “*elephant in the room*”, which led us to modify our analysis plan. In the revised analysis plan, we focus on the analysis of data from sub-cohorts (which are the only studies suggesting a *Gateway effect*) and on the discordance between the data reported by the sub-cohorts and the conclusions of the sub-cohorts. We chose to restrict analyses of non-longitudinal studies to only data useful to characterizing the *Diversion effect* of e-cigarettes on cigarette consumption in adolescents.

The 22 prospective studies have a homogeneous design and have been analyzed by others in 5 recent meta-analyses, whereas the non-prospective studies present designs that are too different to conduct a meta-analysis. This contrasted situation may have contributed to reducing the attention given to these studies where a *Diversion effect* was found.

For longitudinal studies, the investigation of personal and environmental confounders was not conducted as deeply as we originally anticipated because our attention was captured by the major problem of the exclusion of subjects from the analytical samples.

A link exists between e-cigarettes and cigarette initiation in the small, biased subpopulations included in sub-cohorts. We propose in our review a method of estimation on the number of initiations of cigarette use by this trajectory. Even considering, with the conservative hypothesis, that this link is only causal, this is not enough to offset the very strong overall *Diversion effect*.

Estimating the number of excluded smokers to reconstitute whole cohorts is a sensitive point in our analysis. Figures of excluded smokers at T1 are solid when authors provide the data, but in 4/22 studies [33,45,50,53], figures are partially or totally obtained by calculation or estimate from public sources analyzing the same cohorts (3/4) or a similar cohort (1/4) (Supplementary Material Table S5). Nevertheless, the exclusion of these four studies would not substantially alter the extent of the selection of the sub-cohorts and our conclusions.

The cigarette users analyzed in 15/23 publications include only puff experimentation, and only 8/23 publications analyze only cigarette use during the last 30 days (representing 25% of the population studied), but the Khouja et al. meta-analysis [22] report only a marginal variation between the categories of experimenters and users of the e-cigarette.

One other limitation of our study is the subjective scoring by successive votes of only five experts of the authors’ conclusions. One of the main causes of a high score is the dissociation between the conclusions of the experts who report a causal link for the whole cohort between e-cigarette experimentation and cigarette initiation when the results of each sub-cohort concern only a very small, biased fraction of the whole cohort. The limitation is identic for the suggestions to change the regulation of e-cigarettes based on inappropriate extrapolation of sub-cohort results.

## 5. Conclusions

The interactions between e-cigarettes and cigarettes in adolescents are not only one-way. Cross-sectional studies show a decline in tobacco use as well as an increase in e-cigarette use among adolescents, suggesting that e-cigarettes generally turn potential smokers away from tobacco since they first appeared on the market (*Diversion effect*). However, most longitudinal studies conclude the existence of a general *Gateway effect* of e-cigarettes toward cigarette initiation. The discrepancy between the two sources comes mainly from the fact that the longitudinal studies rely on small sub-cohorts of individuals who do not smoke tobacco and may switch from e-cigarettes to cigarettes.

Excluding initial tobacco smokers, the sub-cohorts represent only a small fraction of the whole population: they explain a maximum of 5.3% of tobacco smoking initiation in adolescents, assessing only one way of the interaction (the *Gateway effect*), and exclude any possibility of identifying and quantifying a *Diversion effect* of cigarettes by e-cigarettes.

There is an urge to reconsider the discrepancies between sub-cohort results and published conclusions of longitudinal studies that generalize without coherence and conclude a strong *Gateway effect* of e-cigarettes toward cigarette use in all adolescents and to identify the mechanisms that have led to such scientific dysfunction.

While nicotine abstinence remains the best medical option, over-regulation of e-cigarettes among youth because of misinterpretation of results of longitudinal studies may be detrimental to public health and tobacco control.

## Figures and Tables

**Table 1:** The sub-cohort analyzing effect of e-cigarettes on cigarette initiation after exclusion of all initial smokers (22 studies/23 publications): characteristics of the population.

Authors	Age (Year)	T2-T1 (Month)	Reconstituted Cohort after Reinstating Smokers at T1 (*n*)	Smokers Excluded to Create Sub-Cohort of Non-Smokers at T1 (*n*)	Dual-Users Excluded at T1 (With Smoker) *	Non-Smokers in Analysis at T1 (Included in Sub-Cohort)	Only E-Cigarette at T1	Naïve at T1
Leventhal USA [32]	14.1	6 and 12	3298	768	376	2530	241	2289
Primack USA [33]	20.0	9	788	94		694	16	678
Barrington-Trimis 2016 USA [34]	17.4	16	2078	390	67	1688	146	1542
Wills 2016 (2) TAIWAN [35]	14.8	12	1009	133	135	876	168	708
Best UK [36]	14.4	12	3251	571		2680	183	2497
Hammond CANADA [37]	15.1	12	19,310	1992		17,318	487	16,831
Lozano MEXICO [38]	12.5	20	6006	1311		4695	234	4461
Miech USA [39]	14.1	12	347	101	43	246	11	235
Spindle USA [40]	18.5	12	2996	833	690	2163	153	2010
Wills (1) 2017 USA [41]	14.7	12	1421	351	195	1070	250	820
Aleyan CANADA [42]	15.0	12	11,028	1527		9501	206	9295
Barrington-Trimis 2018 USA [43]	15.5	6 and 12	5041	910		4131	857	3274
Conner UK [26]	13.5	12	2088	318	70	1770	273	1497
East UK [44]	14.5	6	1152	229	111	923	21	902
Loukas USA [45]	19.7	18	4575	2017		2558	558	2000
Morgenstern GERMANY [46]	15.5	6	3667	1141	593	2526	313	2213
Berry USA [47]	13.4	12	6349	1270		5079	527	4552
Chien TAIWAN [48]	14.7	24	15,124	2841		12,283	661	11,622
Kinnunen FINLAND [49]	15.9	24	4454	1299	736	3155	103	3052
Sun UK [50]	14.0	12	12,631	1071		11,560	1306	10,254
Watkins USA [51]	14.3	12	12,106	1612		10,494	425	10,069
Martinelli NEDERLAND [52]	13.6	6 or 12	2803	609	345	2194	191	2003
Osibogun USA [53]	14.5	11	8278	1837		6441	38	6403
Total (all)			129,800	23,225		106,575	7368	99,207
* Total with T1 dual-use data			25,313	6172	3361	1914	1870	17,271

* Concerns 11/23 studies with dual-users’ identification at T1.

**Table 2:** Results (RR, AOR, or OR) and 95% CI of the risk of experimenting with cigarettes or smoking at T2 in non-tobacco smokers at T1 according to experimentation or use of e-cigarettes, compared to non-users (22 studies and 23 publications).

Authors	OR Results (ORa (or OR) and 95% CI) of the Risk of Experimenting with Cigarettes or Smoking at T2 in Non-Tobacco Smokers at T1 According to Experimentation or Use of E-Cigarettes Compared to Non-Users	Main ORa
Leventhal USA [32]	Never-smoker (NS) experimenters with e-cigarettes were more numerous than non-experimenters with e-cigarettes at T1 to (1) have experimented with cigarettes at 6 or 12 months (AOR = 1.75 (1.10–2.77)); (2) have used one or more tobacco products (shisha, cigar, or others) (AOR = 2.73 (2.0–3.73)).	1.75
Primack USA [33]	Due to the small number of e-cigarette users at T1 (*n* = 16), the available results only concern e-cigarette experimenters with no propensity to become smokers (too small number of e-cigarette users with a propensity to become a smoker): e-cigarette experimenters with no propensity to become smokers were more likely to have tried cigarettes 9 months later (AOR = 8.3 (1.2–58.6)) than e-cigarette non-experimenters.	8.3
Barrington-Trimis 2016 USA [34]	Never-smoker users of e-cigarettes, compared to non-users of e-cigarettes, were more likely to have smoked cigarettes without having smoked in the last 30 days at the end of follow-up (AOR = 5.49 (2.68–11.2)) and to have smoked in the past 30 days at the end of follow-up (AOR = 7.50 (2.41–23.4)) if they had used e-cigarettes at T1, but with a lower AOR than in the non-susceptible (AOR = 2.58 (1.30–5.09)).	5.49
Wills 2016 (2) TAI- WAN [35]	E-cigarette experimenters, compared to e-cigarette non-experimenters, were less at risk of having tried cigarettes at T2 when the percentile of the propensity to become a smoker was high: (1) percentile 10 (AOR = 2.23 (1.57–3.17)); (2) percentile 50 (AOR = 1.76 (1.47–2.10)); and (3) percentile 90 (AOR = 1.32 (1.19–1.47)).	1.76
Best UK [36]	More young adults who used e-cigarettes at T1 had tried cigarettes at T2 (12 months) (AOR = 2.42 (1.63–3.60)) than non-users of e-cigarettes at T1.	2.42
Hammond CANADA [37]	Non-smokers who had used e-cigarettes in the past 30 days were more likely than non-users of e-cigarettes in the past 30 days to (1) have smoked at least one cigarette before T2 (AOR = 2.12 (1.68–2.66)) and (2) be a daily smoker at T2 (AOR = 1.79: (1.41–2.28)).	2.12
Lozano MEXICO [38]	E-cigarette experimenters were more likely to experiment with cigarettes at T2 (20 months later) (adjusted RR = 1.41 (1.18–1.70)) than e-cigarette non-experimenters. E-cigarette experimenters were not significantly more likely to experiment with marijuana at T2 (RRa = 1.42 (0.84–2.37)) than e-cigarette non-experimenters. On the other hand, those who had tried tobacco cigarettes were more likely to have tried marijuana (RRa = 2.05 (1.53–2.75)).	1.41
Miech USA [39]	Never-smokers experimenting with e-cigarettes were more likely to smoke cigarettes 12 months later (RR = 4.78 (1.91–11.96)) than non-experimenters of e-cigarettes.	4.78
Spindle USA [40]	Non-smokers experimenting with e-cigarettes were more likely to have experimented with cigarettes 12 months later (AOR = 3.30 (20–9.05)) than non-experimenters of e-cigarettes. Conversely, young people who have used e-cigarettes in the last 30 days were not significantly more likely to have tried cigarettes 12 months later (AOR = 1.15 (0.15–9.06)) than non-users of e-cigarettes in the past 30 days.	3.3
Wills (1) 2017 USA [41]	Non-smokers who used e-cigarettes were more likely to have experimented with cigarettes at T2 (12 months later) (AOR = 2.87 (2.03–4.05)) than non-experimenters of e-cigarettes. Non-smokers who used e-cigarettes at least several times a week were more likely to have tried cigarettes at T2 (AOR = 4.09 (2.43–6.88)) than e-cigarette non-experimenters.	2.87
Aleyan CANADA [42]	Young people with non-susceptibility to become tobacco smokers at T1 were more likely to have tried tobacco cigarettes at T2 (OR = 5.28; (2.81–9.94)). Young people susceptible at T1 were more likely to have tried tobacco cigarettes at T2 (OR = 2.78 (1.84–4.20)); *p* < 0.001, but with a lower OR than the non-susceptible if they were current e-cigarette users at T1.	5.28
Barrington-Trimis 2018 USA [43]	E-cigarette users were more likely to have tried cigarettes at the end of follow-up (OR = 4.58 (3.56–5.88)) or to have smoked more than three cigarettes in the last 30 days at T2 (OR = 3.51 (1.97–6.24)) than non-e-cigarette users.	4.58
Conner UK [26]	Baseline ever use of e-cigarettes was strongly associated with subsequent initiation of cigarettes (AOR = 4.6 (2.94–5.60)) but not with escalated cigarette use (AOR = 1.9 (0.97–1.82)).	4.06
East UK [44]	E-cigarette use at any point is associated with an AOR = 3.54 (1.68–7.45) to become a smoker compared to e-cigarette use at no point. But there is a bilateral effect: direct effect of e-cigarettes on smoking initiation (AOR = 1.34 (1.05–1.72)) and a direct effect on cigarette use at any point on e-cigarette initiation (AOR = 1.08 (1.01–1.17)).	3.54
Loukas USA [45]	E-cigarette experimenters were more likely to have experimented with cigarettes at T2 (18 months later) (AOR = 1.36 (1.01–1.83)) than non-experimenters of e-cigarettes. The AOR was higher in exclusive e-cigarette experimenters (AOR = 2.26 (2.35–3.76)), but it was not significant in those who had experimented with other tobacco products (shisha, cigar, etc.) in addition to e-cigarettes (AOR = 1.13 (0.81–1.58)).	2.26
Morgen- stern GER- MANY [46]	E-cigarette experimenters were more likely to have tried cigarettes at T2 (6 months later) (RR = 2.18 (1.65–2.83)) than e-cigarette non-experimenters.	2.18
Berry USA [47]	When the first product used was an e-cigarette, an increased risk was observed to (vs. tobacco product or e-cigarette use at no point) (1) experiment with tobacco cigarettes before T2 (AOR = 4.09 (2.97–5.63)) and (2) have smoked in the last 30 days before T2 (AOR = 2.75 (1.60–4.73)). Among e-cigarette experimenters, the risk of experimenting with cigarettes was higher for e-cigarette users with no propensity to become a smoker (AOR = 8.57 (3.87–18.97)) than for high propensity group (AOR = 3.51 (2.52–4.89)).	4.09
Chien TAI- WAN [48]	E-cigarette experimenters were more likely to have tried cigarettes at the end of follow-up (AOR = 2.14 (1.66–2.75)) than e-cigarette non-experimenters.	2.14
Kinnunen FINLAND [49]	Among T1 never-smokers, (1) experimentation or use of nicotine-containing e-cigarettes predicted the uptake of daily smoking at T2 (AOR = 2.92 (1.09–7.85)) and (2) T1 experimentation with non-nicotine e-cigarettes did not predict the uptake of daily smoking at T2 (small sample size).	2.92
Sun UK [50]	E-cigarette experimenters were more likely to have tried cigarettes 12 months later than e-cigarette non-experimenters for the 2014 wave (AOR = 2.10 (1.33–3.30)), 2015 wave (AOR = 2.09 (1.26–3.48)), and 2016 wave (AOR = 2.25 (1.55–3.27)). The effect of e-cigarettes was no longer significant in the 2017 wave (AOR = 1.40 (0.91–2.14)) and the 2018 wave (AOR = 1.35 (0.84–2.16)).	2.1
Watkins USA [51]	Non-smoker experimenters of e-cigarettes at T1 were more likely to have experimented with cigarettes at T2 (AOR = 2.53 (1.80–3.56)) than non-experimenters of e-cigarettes as well as e-cigarette users in the last 30 days (AOR = 1.87 (1.15–3.05)). Non-smokers who experimented exclusively with e-cigarettes were more likely to have tried cigarettes at T2 (AOR = 2.99 (1.98–4.53)), as e-cigarette users in the past 30 days (AOR = 2.12 (1.11–4.03)). Compared to the use of any tobacco product other than e-cigarettes, poly-consumption of tobacco products at T1 increased the probability at T2 of initiating smoking (AOR = 3.95 (2.65–5.90)) and to cigarette 30-day use (AOR = 3.81 (2.22–6.54)).	2.53
Martinelli NEDER- LAND [52]	The *Gateway effect* goes in both directions: (1) in a never-smoker at T1, use of e-cigarettes was associated with an AOR = 5.63 (3.04–10.42) to use cigarettes at T2, (2) smoking at T1 is associated with an AOR = 3.10 (1.58–6.06) to e-cigarette use at T2.	5.63
Osibogun USA [53]	Using the e-cigarette at T1 is associated with an increased risk of experimenting with cigarettes in non-smokers (AOR = 5.0 (1.9–12.8)).	5
	Mean	3.5
	median	2.92
	min	1.41
	maxi	8.3

**Table 3:** Main results of the 5 meta-analyses of sub-cohorts analyzing the effect of e-cigarettes on cigarette initiation after exclusion of initial smokers.

Authors [Reference] and Number of Studies in Meta-Analysis	OR (95% CI)	AOR (95% CI)	Heterogeneity (I2) for AOR	AOR Fully Adjusted (See Notes)	(95% CI)	Heterogeneity (I2) for AOR
	3.83 (3.74–3.91)	3.5 (2.38–5.16)	56%	3.16 *	(2.14–4.66)	
Soneji [21]				3.03 ¥	(1.65–5.55)	80%
7				4.11 ¥¥	(2.63–6.41)	
				2.77 §	(1.67–4.60)	52%
				4.48 §§	(3.06–6.57)	
	4.17 (3.53–6.29) °	3.13 (2.35–4.16)	84%			
Khouja [22]	4.35 (2.95–6.42) °°	2.21 (1.72–2.84)	5%			
14	4.59 (3.60–5.85) °°°	2.92 (2.30–3.71)	85%			
	4.31 (3.33–5.58)	2.93 (2.22–3.87)	84%			
Chan [23]				6.68 #	(3.63–12.31)	
11				2.49 ##	(1.97–3.15)	
	4.81 (3.79–6.12)	4.06 (3.00–5.48)	68%	2.81 §	(2.45–3.72)	78%
O’Brien [24]		3.71 (2.83–4.86)	35%	5.16 §§	(3.69–7.21)	38%
9				11.32 ~	(5.35–23.95)	0%
				3.54 ~~	(2.70–4.65)	62%
				3.18 {	(2.26–4.47)	65%
				6.22 {{	(3.73–10.38)	54%
Yoong [25] 17	3.44 (2.91–4.08)	3.01 (2.37–3.82)	82%			

Notes: # model considering studies with a population <1000. ° model considering cigarette use at any point T2. ## model considering studies with a population >1000. °° model considering current use of cigarettes T2. § model considering studies published before 2014. °°° model considering e-cigarette use T1. §§ model considering studies published after 2014 and only considering the 4 high-quality studies. ~ model considering studies with follow-up <12 months. * model adjusted for COPAS selection bias. ~~ model considering studies with follow-up >12 months. ¥ model considering subjects <18 years old. { model considering North American studies. ¥¥ model considering subjects ≥18 years old. {{ model taking into account European studies.

**Table 4:** Distribution of the causality score and score of requests to change the e-cigarette regulation in the 23 publications of the 22 cohorts in main analysis.

Score of Requests to Change the E-Cigarette Regulation						
		0	1	2	3	Total
causality score	0	**①**				1
	1	**①**				1
	2	③❷	**①**	❹	❸	13
	3			①❸	❸ + 1	8
	total	7	1	8	7	23

In circles: number of studies published in two continents or in Asia for the designated score. Note: number of studies published for the designated score according to the place of survey data collection. In plain circles: North America (USA/Canada + Mexico); in hollow circles: Europe; and without circle: Taiwan.

## Data Availability

All the data used in this article come from the referenced studies and we specify the origin of each of them in the text or tables.

## Supplementary Materials

The following supporting information can be downloaded at https://www.mdpi.com/article/10.3390/ijerph20206936/s1. Table S1: Prisma checklist. Table S2: Medline query for initial selection of articles to include in the general review. Table S3: Scoring of causal effect of e-cigarette experimentation or use at T1 on cigarette consumption at T2 and scoring of the authors’ general recommendations to restrict use of e-cigarettes in the general population of teenagers or young adults. Table S4: Type of consumption (only experimentation, use, and daily use) of e-cigarettes at T1 and of cigarettes at T2. Table S5: Sources of the figures used to recalculate the number of smokers initially excluded in the 22 cohorts. Table S6: Additional information on populations included at T1 and assessed for cigarette experimentation or use at T2 used to constitute and analyze the 22 sub-cohorts. Table S7: Detail of progressive consensus of 5 experts with 5 successive votes to reach an agreement on the scoring of authors on the causality (*Gateway effect* of e-cigarettes on cigarette initiation in adolescents) and on the proposal to reinforce regulation and to decrease attractivity of the e-cigarette. Table S8: Influence of first product experimented (cigarette or e-cigarette) on the subsequent consumption of the other.

## References

[1] Proctor R. (2011). Golden Holocaust: Origins of the Cigarette Catastrophe and the Case for Abolition.

[2] Sharapova S., Reyes-Guzman C., Singh T., Phillips E., Marynak K.L., Agaku I. (2020). Age of tobacco use initiation and association with current use and nicotine dependence among US middle and high school students, 2014–2016. Tob. Control.

[3] Grana R.A. (2013). Electronic cigarettes: A new nicotine gateway?. J. Adolesc. Health.

[4] Chapman S., Bareham D., Maziak W. (2019). The Gateway Effect of E-cigarettes: Reflections on Main Criticisms. Nicotine Tob. Res..

[5] Glasser A., Abudayyeh H., Cantrell J., Niaura R. (2019). Patterns of E-Cigarette Use Among Youth and Young Adults: Review of the Impact of E-Cigarettes on Cigarette Smoking. Nicotine Tob. Res..

[6] Case K.R., Obinwa U.C., Clendennen S.L., Perry C.L., Harrell M.B. (2020). Predictors of JUUL, other electronic nicotine delivery systems, and combustible tobacco initiation among Texas youth. Prev. Med..

[7] Loukas A., Marti C.N., Harrell M.B. (2022). Electronic nicotine delivery systems use predicts transitions in cigarette smoking among young adults. Drug Alcohol Depend..

[8] Audrain-McGovern J., Rodriguez D., Pianin S., Testa S. (2021). Conjoint Developmental Trajectories of Adolescent E-cigarette and Combustible Cigarette Use. Pediatrics.

[9] Ferkol T.W., Farber H.J., La Grutta S., Leone F.T., Marshall H.M., Neptune E., Pisinger C., Vanker A., Wisotzky M., Zabert G.E. (2018). Electronic cigarette use in youths: A position statement of the Forum of International Respiratory Societies. Forum of International Respiratory Societies. Eur. Respir. J..

[10] Polosa R., Casale T.B., Tashkin D.P. (2022). A Close Look at Vaping in Adolescents and Young Adults in the United States. J. Allergy Clin. Immunol. Pract..

[11] Gmel G., Baggio S., Mohler-Kuo M., Daeppen J.B., Studer J. (2016). E-cigarette use in young Swiss men: Is vaping an effective way of reducing or quitting smoking?. Swiss Med. Wkly..

[12] Mantey D.S., Cooper M.R., Loukas A., Perry C.L. (2017). E-cigarette use and cigarette smoking cessation among texas college students. Am. J. Health Behav..

[13] Lindson N., Theodoulou A., Ordóñez-Mena J.M., Fanshawe T.R., Sutton A.J., Livingstone-Banks J., Hajizadeh A., Zhu S., Aveyard P., Freeman S.C. (2023). Pharmacological and electronic cigarette interventions for smoking cessation in adults: Component network meta-analyses. Cochrane Database Syst. Rev..

[14] Dautzenberg B. (2013). Rapport et Avis D’experts sur L’e-Cigarette Documentation Française.

[15] Park-Lee E., Ren C., Cooper M., Cornelius M., Jamal A., Cullen K.A. (2022). Tobacco Product Use among Middle and High School Students—United States, 2022. Morb. Mortal. Wkly. Rep..

[16] OFDT Les Drogues à 17 Ans—Analyse de L’enquête ESCAPAD 2022. Tendances 223, pp. 1–9. https://www.ofdt.fr/publications/collections/tendances/les-drogues-17-ans-analyse-de-lenquete-escapad-2022-tendances-155-mars-2022/.

[17] WHO (2023). WHO Report on the Global Tobacco Epidemic, 2023: Protect People from Tobacco Smoke.

[18] Etter J.F. (2018). Gateway effects and electronic cigarettes. Addiction.

[19] Selya A.S., Foxon F. (2021). Trends in electronic cigarette use and conventional smoking: Quantifying a possible ‘diversion’ effect among US adolescents. Addiction.

[20] Chatterjee K., Alzghoul B., Innabi A., Meena N. (2018). Is vaping a gateway to smoking: A review of the longitudinal studies. Int. J. Adolesc. Med. Health.

[21] Soneji S., Barrington-Trimis J.L., Wills T.A., Leventhal A.M., Unger J.B., Gibson L.A., Yang J., Primack B.A., Andrews J.A., Miech R.A. (2017). Association Between Initial Use of e-Cigarettes and Subsequent Cigarette Smoking Among Adolescents and Young Adults: A Systematic Review and Meta-analysis. JAMA Pediatr..

[22] Khouja J.N., Suddell S.F., Peters S.E., Taylor A.E., Munafò M.R. (2020). Is e-cigarette use in non-smoking young adults associated with later smoking? A systematic review and meta-analysis. Tob. Control.

[23] Chan G.C.K., Stjepanovic D., Lim C., Sun T., Shanmuga Anandan A., Connor J.P., Gartner C., Hall W.D., Leung J. (2021). Gateway or common liability? A systematic review and meta-analyses of studies of adolescent e-cigarette use and future smoking initiation. Addiction.

[24] O’Brien D., Long J., Quigley J., Lee C., McCarthy A., Kavanagh P. (2021). Association between electronic cigarette use and tobacco cigarette smoking initiation in adolescents: A systematic review and meta-analysis. BMC Public Health.

[25] Yoong S.L., Hall A., Turon H., Stockings E., Leonard A., Grady A., Tzelepis F., Wiggers J., Gouda H., Fayokun R. (2021). Association between electronic nicotine delivery systems and electronic non-nicotine delivery systems with initiation of tobacco use in individuals aged < 20 years. A systematic review and meta-analysis. PLoS ONE.

[26] Conner M., Grogan S., Simms-Ellis R., Flett K., Sykes-Muskett B., Cowap L., Lawton R., Armitage C.J., Meads D., Torgerson C. (2017). Do electronic cigarettes increase cigarette smoking in UK adolescents? Evidence from a 12-month prospective study. Tob. Control.

[27] Kang H., Cho S.I. (2020). Longitudinal transitions of cigarettes and electronic nicotine delivery systems among adolescents: Construction of a retrospective cohort using recall data from a cross-sectional sample. Tob. Induc. Dis..

[28] Chaffee B.W., Watkins S.L., Glantz S.A. (2018). Electronic Cigarette Use and Progression From Experimentation to Established Smoking. Pediatrics.

[29] Sutfin E.L., Reboussin B.A., Debinski B., Wagoner K.G., Spangler J., Wolfson M. (2015). The Impact of trying electronic cigarettes on cigarette smoking by college students: A Prospective analysis. Am. J. Public Health.

[30] Walker N., Parag V., Wong S.F., Youdan B., Broughton B., Bullen C., Beaglehole R. (2020). Use of e-cigarettes and smoked tobacco in youth aged 14-15 years in New Zealand: Findings from repeated cross-sectional studies (2014–2019). Lancet Public Health.

[31] Hallingberg B., Maynard O.M., Bauld L., Brown R., Gray L., Lowthian E., MacKintosh A.M., Moore L., Munafo M.R., Moore G. (2020). Have e-cigarettes renormalised or displaced youth smoking? Results of a segmented regression analysis of repeated cross sectional survey data in England, Scotland and Wales. Tob. Control.

[32] Leventhal A.M., Strong D.R., Kirkpatrick M.G., Unger J.B., Sussman S., Riggs N.R., Stone M.D., Khoddam R., Samet J.M., Audrain-McGovern J. (2015). Association of Electronic Cigarette Use with Initiation of Combustible Tobacco Product Smoking in Early Adolescence. JAMA.

[33] Primack B.A., Soneji S., Stoolmiller M., Fine M.J., Sargent J.D. (2015). Progression to Traditional Cigarette Smoking After Electronic Cigarette Use Among US Adolescents and Young Adults. JAMA Pediatr..

[34] Barrington-Trimis J.L., Urman R., Berhane K., Unger J.B., Cruz T.B., Pentz M.A., Samet J.M., Leventhal A.M., McConnell R. (2016). E-Cigarettes and Future Cigarette Use. Pediatrics.

[35] Wills T.A., Sargent J.D., Gibbons F.X., Pagano I., Schweitzer R. (2016). E-cigarette use is differentially related to smoking onset among lower risk adolescents. Tob. Control.

[36] Best C., Haseen F., Currie D., Ozakinci G., MacKintosh A.M., Stead M., Eadie D., MacGregor A., Pearce J., Amos A. (2017). Relationship between trying an electronic cigarette and subsequent cigarette experimentation in Scottish adolescents: A cohort study. Tob. Control.

[37] Hammond D., Reid J.L., Cole A.G., Leatherdale S.T. (2017). Electronic cigarette use and smoking initiation among youth: A longitudinal cohort study. CMAJ.

[38] Lozano P., Barrientos-Gutierrez I., Arillo-Santillan E., Morello P., Mejia R., Sargent J.D., Thrasher J.F. (2017). A longitudinal study of electronic cigarette use and onset of conventional cigarette smoking and marijuana use among Mexican adolescents. Drug Alcohol Depend..

[39] Miech R., Patrick M.E., O’Malley P.M., Johnston L.D. (2017). E-cigarette use as a predictor of cigarette smoking: Results from a 1-year follow-up of a national sample of 12th grade students. Tob. Control.

[40] Spindle T.R., Hiler M.M., Cooke M.E., Eissenberg T., Kendler K.S., Dick D.M. (2017). Electronic cigarette use and uptake of cigarette smoking: A longitudinal examination of U.S. college students. Addict. Behav..

[41] Wills T.A., Knight R., Sargent J.D., Gibbons F.X., Pagano I., Williams R.J. (2017). Longitudinal study of e-cigarette use and onset of cigarette smoking among high school students in Hawaii. Tob. Control.

[42] Aleyan S., Cole A., Qian W., Leatherdale S.T. (2018). Risky business: A longitudinal study examining cigarette smoking initiation among susceptible and non-susceptible e-cigarette users in Canada. BMJ Open.

[43] Barrington-Trimis J.L., Kong G., Leventhal A.M., Liu F., Mayer M., Cruz T.B., Krishnan-Sarin S., McConnell R. (2018). E-cigarette Use and Subsequent Smoking Frequency Among Adolescents. Pediatrics.

[44] East K., Hitchman S.C., Bakolis I., Williams S., Cheeseman H., Arnott D., McNeill A. (2018). The Association Between Smoking and Electronic Cigarette Use in a Cohort of Young People. J. Adolesc. Health.

[45] Loukas A., Marti C.N., Cooper M., Pasch K.E., Perry C.L. (2018). Exclusive e-cigarette use predicts cigarette initiation among college students. Addict. Behav..

[46] Morgenstern M., Nies A., Goecke M., Hanewinkel R. (2018). E-Cigarettes and the Use of Conventional Cigarettes. Dtsch. Arztebl. Int..

[47] Berry K.M., Fetterman J.L., Benjamin E.J., Bhatnagar A., Barrington-Trimis J.L., Leventhal A.M., Stokes A. (2019). Association of Electronic Cigarette Use With Subsequent Initiation of Tobacco Cigarettes in US Youths. JAMA Netw. Open.

[48] Chien Y.N., Gao W., Sanna M., Chen P.L., Chen Y.H., Glantz S., Chiou H.Y. (2019). Electronic Cigarette Use and Smoking Initiation in Taiwan: Evidence from the First Prospective Study in Asia. Int. J. Environ. Res. Public Health.

[49] Kinnunen J.M., Ollila H., Minkkinen J., Lindfors P.L., Timberlake D.S., Rimpelä A.H. (2019). Nicotine matters in predicting subsequent smoking after e-cigarette experimentation: A longitudinal study among Finnish adolescents. Drug Alcohol Depend..

[50] Sun R., Mendez D., Warner K.E. (2022). Is Adolescent E-Cigarette Use Associated With Subsequent Smoking? A New Look. Nicotine Tob. Res..

[51] Watkins S.L., Glantz S.A., Chaffee B.W. (2018). Association of Noncigarette Tobacco Product Use With Future Cigarette Smoking Among Youth in the Population Assessment of Tobacco and Health (PATH) Study, 2013-2015. JAMA Pediatr..

[52] Martinelli T., Candel M.J.J.M., de Vries H., Talhout R., Knapen V., van Schayck C.P., Nagelhout G.E. (2023). Exploring the gateway hypothesis of e-cigarettes and tobacco: A prospective replication study among adolescents in the Netherlands and Flanders. Tob. Control.

[53] Osibogun O., Bursac Z., Maziak W. (2020). E-Cigarette Use and Regular Cigarette Smoking Among Youth: Population Assessment of Tobacco and Health Study (2013–2016). Am. J. Prev. Med..

[54] Wills T.A., Gibbons F.X., Sargent J.D., Schweitzer R.J. (2016). How is the effect of adolescent e-cigarette use on smoking onset mediated: A longitudinal analysis. Psychol. Addict. Behav..

[55] Penzes M., Foley K.L., Nădășan V., Paulik E., Ábrám Z., Urbán R. (2018). Bidirectional associations of e-cigarette, conventional cigarette and waterpipe experimentation among adolescents: A cross-lagged model. Addict. Behav..

[56] Bold K.W., Kong G., Camenga D.R., Simon P., Cavallo D.A., Morean M.E., Krishnan-Sarin S. (2018). Trajectories of e-cigarette and conventional cigarette use among youth. Pediatrics.

[57] Staff J., Kelly B.C., Maggs J.L., Vuolo M. (2022). Adolescent electronic cigarette use and tobacco smoking in the Millennium Cohort Study. Addiction.

[58] Aleyan S., Gohari M.R., Cole A.G., Leatherdale S.T. (2019). Exploring the Bi-Directional Association between Tobacco and E-Cigarette Use among Youth in Canada. Int. J. Environ. Res. Public Health.

[59] Foxon F., Selya A.S. (2020). Electronic cigarettes, nicotine use trends and use initiation ages among US adolescents from 1999 to 2018. Addiction.

[60] Levy D.T., Warner K.E., Cummings K.M., Hammond D., Kuo C., Fong G.T., Thrasher J.F., Goniewicz M.L., Borland R. (2019). Examining the relationship of vaping to smoking initiation among US youth and young adults: A reality check. Tob. Control.

[61] Vogel E.A., Cho J., McConnell R.S., Barrington-Trimis J.L., Leventhal A.M. (2020). Prevalence of Electronic Cigarette Dependence Among Youth and Its Association With Future Use. JAMA Netw. Open.

[62] Legleye S., Aubin H.J., Falissard B., Beck F., Spilka S. (2021). Experimenting first with e-cigarettes versus first with cigarettes and transition to daily cigarette use among adolescents: The crucial effect of age at first experiment. Addiction.

[63] Chyderiotis S., Benmarhnia T., Beck F., Spilka S., Legleye S. (2020). Does e-cigarette experimentation increase the transition to daily smoking among young ever-smokers in France?. Drug Alcohol Depend..

[64] Doran N., Brikmanis K., Petersen A., Delucchi K., Al-Delaimy W.K., Luczak S., Myers M., Strong D. (2017). Does e-cigarette use predict cigarette escalation? A longitudinal study of young adult non-daily smokers. Prev. Med..

[65] Cardenas V.M., Evans V.L., Balamurugan A., Faramawi M.F., Delongchamp R.R., Wheeler J.G. (2016). Use of electronic nicotine delivery systems and recent initiation of smoking among US youth. Int. J. Public Health.

[66] Sokol N.A., Feldman J.M. (2021). High School Seniors Who Used E-Cigarettes May Have Otherwise Been Cigarette Smokers: Evidence From Monitoring the Future (United States, 2009–2018). Nicotine Tob. Res..

[67] Kinnunen J.M., Ollila H., Minkkinen J., Lindfors P.L., Rimpelä A.H. (2018). A Longitudinal Study of Predictors for Adolescent Electronic Cigarette Experimentation and Comparison with Conventional Smoking. Int. J. Environ. Res. Public Health.

[68] Do E.K., Tulsiani S., Vallone D.M., Hair E.C. (2022). Transitions in Frequent to Daily Tobacco and Nicotine Use among Youth and Young Adults. Subst. Use Misuse.

[69] Pierce J.P., Chen R., Leas E.C., White M.M., Kealey S., Stone M.D., Benmarhnia T., Trinidad D.R., Strong D.R., Messer K. (2021). Use of E-cigarettes and Other Tobacco Products and Progression to Daily Cigarette Smoking. Pediatrics.

[70] Shahab L., Beard E., Brown J. (2021). Association of initial e-cigarette and other tobacco product use with subsequent cigarette smoking in adolescents: A cross-sectional, matched control study. Tob. Control.

[71] Mus S., Monzon J., Islam F., Thrasher J.F., Barnoya J. (2023). First tobacco product tried and current use of cigarettes and electronic cigarettes among adolescents from Guatemala City. Salud Pública México.

[72] McCabe S.E., Veliz P., McCabe V.V., Boyd C.J. (2019). Initiation Sequence of E-Cigarette and Cigarette Smoking among US Adolescents: A National Study. Am. J. Addict..

[73] Evans-Polce R.J., Veliz P., Boyd C.J., McCabe S.E. (2020). Initiation Patterns and Trends of E-Cigarette and Cigarette Use among U.S. Adolescents. J. Adolesc. Health.

[74] Notley C., Gentry S., Cox S., Dockrell M., Havill M., Attwood A.S., Smith M., Munafò M.R. (2022). Youth use of e-liquid flavours-a systematic review exploring patterns of use of e-liquid flavours and associations with continued vaping, tobacco smoking uptake or cessation. Addiction.

[75] Friedman A.S., Xu S. (2020). Associations of Flavored e-Cigarette Uptake With Subsequent Smoking Initiation and Cessation. JAMA Netw. Open.

[76] Groom A.L., Vu T.T., Kesh A., Hart J.L., Walker K.L., Giachello A.L., Sears C.G., Tompkins L.K., Mattingly D.T., Landry R.L. (2020). Correlates of youth vaping flavor preferences. Prev. Med. Rep..

[77] Harrell M.B., Weaver S.R., Loukas A., Creamer M., Marti C.N., Jackson C.D., Heath J.W., Nayak P., Perry C.L., Pechacek T.F. (2017). Flavored e-cigarette use: Characterizing youth, young adult, and adult users. Prev. Med. Rep..

[78] Goldenson N.I., Leventhal A.M., Stone M.D., McConnell R.S., Barrington-Trimis J.L. (2017). Associations of electronic cigarette nicotine concentration with subsequent cigarette smoking and vaping levels in adolescents. JAMA Pediatr..

[79] Auf R., Trepka M.J., Selim M., Ben Taleb Z., De La Rosa M., Bastida E., Cano M.Á. (2019). E-cigarette use is associated with other tobacco use among US adolescents. Int. J. Public Health.

[80] Treur J.L., Rozema A.D., Mathijssen J.J.P., van Oers H., Vink J.M. (2018). E-cigarette and waterpipe use in two adolescent cohorts: Cross-sectional and longitudinal associations with conventional cigarette smoking. Eur. J. Epidemiol..

[81] Evans-Polce R., Veliz P., Boyd C.J., McCabe V.V., McCabe S.E. (2020). Trends in E-Cigarette, Cigarette, Cigar, and Smokeless Tobacco Use Among US Adolescent Cohorts, 2014-2018. Am. J. Public Health.

[82] Huh J., Leventhal A.M. (2016). Progression of Poly-tobacco Product Use Patterns in Adolescents. Am. J. Prev. Med..

[83] Stanton C.A., Sharma E., Seaman E.L., Kasza K.A., Edwards K.C., Halenar M.J., Taylor K.A., Day H., Anic G., Hull L.C. (2020). Initiation of any tobacco and five tobacco products across 3 years among youth, young adults and adults in the USA: Findings from the PATH Study Waves 1–3 (2013–2016). Tob. Control.

[84] Campus B., Fafard P., St Pierre J., Hoffman S.J. (2021). Comparing the regulation and incentivization of e-cigarettes across 97 countries. Soc. Sci. Med..

[85] Xu S., Coffman D.L., Liu B., Xu Y., He J., Niaura R.S. (2022). Relationships Between E-cigarette Use and Subsequent Cigarette Initiation Among Adolescents in the PATH Study: An Entropy Balancing Propensity Score Analysis. Prev. Sci..

[86] Kim S., Selya A.S. (2020). The Relationship Between Electronic Cigarette Use and Conventional Cigarette Smoking Is Largely Attributable to Shared Risk Factors. Nicotine Tob. Res..

[87] Hughes J., Sykes G., Hughes K., O’Reilly M., Goodwin J., Sutton C., Karim K. (2021). From gateways to multilinear connections: A qualitative longitudinal investigation of the relationships between vaping and smoking among adolescent users. Int. J. Drug Policy.

[88] Wang M.P., Li W.H., Wu Y., Lam T.H., Chan S.S. (2017). Electronic cigarette use is not associated with quitting of conventional cigarettes in youth smokers. Pediatr. Res..

[89] Hartmann-Boyce J., McRobbie H., Butler A.R., Lindson N., Bullen C., Begh R., Theodoulou A., Notley C., Rigotti N.A., Turner T. (2021). Electronic cigarettes for smoking cessation. Cochrane Database Syst. Rev..

[90] Al-Hamdani M., Manly E. (2021). Smoking cessation or initiation: The paradox of vaping. Prev. Med. Rep..

