# Supplementary material for: Systematic Review and Critical Analysis of Longitudinal Studies Assessing Effect of E-Cigarettes on Cigarette Initiation among Adolescent Never-Smokers
Source: Int J Environ Res Public Health. 2023 Oct 18;20(20):6936. doi: 10.3390/ijerph20206936 (PMC10606427; doi:10.3390/ijerph20206936)
Supplement: Supplementary file 1 [file ijerph-20-06936-s001.zip › ijerph-2598062-supplementary.pdf]

Supplementary Materials: The following supporting information can be downloaded at: www.mdpi.com/xxx/s1,

Table S1. Prisma Check list.

Table S2. Medline Query for initial selection of articles to include in the general review.

Table S3. Scoring of causal effect of e-cigarettes experimentation or use at T1 on the cigarette consumption at T2 and scoring of the authors general recommendations to restrict use of e-cigarette in the general population of teenagers or young adults.

Table S4. Type of consumption (only experimentation, use, daily use) of e-cigarettes at T1 and of cigarettes at T2.

Table S5. Sources of the figures used to recalculate the number of smokers initially excluded in the 22 cohorts.

Table S6. Additional information on populations included at T1 and assessed for cigarette experimentation or use at T2 used to constitute and analyze the 22 sub-cohorts.

Table S7. Detail of progressive consensus of 5 experts with 5 successive votes to reach an agreement on the scoring of authors on the causality (gateway effect of e-cigarette on cigarette initiation in adolescents) and on the proposal to reinforce regulation and to decreasing attractivity of the e-cigarette.

Table S8. Influence of first product experimented (cigarette or e-cigarette) on the subsequent consumption of the other.

Table S1. Prisma Check list.t

Section and Topic	Item #	Checklist item	Location where item is reported
**TITLE**			
Title	1	Identify the report as a systematic review.	Page 2
**ABSTRACT**			
Abstract	2	See the PRISMA 2020 for Abstracts checklist.	Abstract Sup S1
**INTRODUCTION**			
Rationale	3	Describe the rationale for the review in the context of existing knowledge.	P&-2 Flow chart Figure 1
Objectives	4	Provide an explicit statement of the objective(s) or question(s) the review addresses.	Introduction/discussion/conclusion
**METHODS**			
Eligibility criteria	5	Specify the inclusion and exclusion criteria for the review and how studies were grouped for the syntheses.	Flow chart Figure 1, &2.2 &2.3
Information sources	6	Specify all databases, registers, websites, organisations, reference lists and other sources searched or consulted to identify studies. Specify the date when each source was last searched or consulted.	Flow chart (Figure 1) Tabl 2
Search strategy	7	Present the full search strategies for all databases, registers and websites, including any filters and limits used.	Sup S2
Selection process	8	Specify the methods used to decide whether a study met the inclusion criteria of the review, including how many reviewers screened each record and each report retrieved, whether they worked independently, and if applicable, details of automation tools used in the process.	&2.3
Data collection process	9	Specify the methods used to collect data from reports, including how many reviewers collected data from each report, whether they worked independently, any processes for obtaining or confirming data from study investigators, and if applicable, details of automation tools used in the process.	&2.3
Data items	10a	List and define all outcomes for which data were sought. Specify whether all results that were compatible with each outcome domain in each study were sought (e.g. for all measures, time points, analyses), and if not, the methods used to decide which results to collect.	&2.43 &2.4.3 &2.5.1
	10b	List and define all other variables for which data were sought (e.g. participant and intervention characteristics, funding sources). Describe any assumptions made about any missing or unclear information.	Table 1 table 2 &2.3 &2.4.2 &2.4.3 sup S4, S5, S6
Study risk of bias assessment	11	Specify the methods used to assess risk of bias in the included studies, including details of the tool(s) used, how many reviewers assessed each study and whether they worked independently, and if applicable, details of automation tools used in the process.	Page 2, 7-8,14,15 Figure 3 et 4 Sup S4 ,S6 &4.2.4.3 &4.2.4.5
Effect measures	12	Specify for each outcome the effect measure(s) (e.g. risk ratio, mean difference) used in the synthesis or presentation of results.	Page 7, 8, 11, &2.4.1 &2.4.2 &3.2 Table 2, Figure 4, Sup S4, S5
Synthesis methods	13a	Describe the processes used to decide which studies were eligible for each synthesis (e.g. tabulating the study intervention characteristics and comparing against the planned groups for each synthesis (item #5)).	Figure 1 Table 1

	13b	Describe any methods required to prepare the data for presentation or synthesis, such as handling of missing summary statistics, or data conversions.	Sup S4, S5, Page 3 Figure 4
	13c	Describe any methods used to tabulate or visually display results of individual studies and syntheses.	Table 1, Table 2, Supplementary material S4, S5, S6, Figure 4
	13d	Describe any methods used to synthesize results and provide a rationale for the choice(s). If meta-analysis was performed, describe the model(s), method(s) to identify the presence and extent of statistical heterogeneity, and software package(s) used.	NA: No new meta-analysis
	13e	Describe any methods used to explore possible causes of heterogeneity among study results (e.g. subgroup analysis, meta-regression).	Using only others authors meta-analyse results T3
	13f	Describe any sensitivity analyses conducted to assess robustness of the synthesized results.	Sup S4, S5, S6
Reporting bias assessment	14	Describe any methods used to assess risk of bias due to missing results in a synthesis (arising from reporting biases).	Sup S5
Certainty assessment	15	Describe any methods used to assess certainty (or confidence) in the body of evidence for an outcome.	NA
**RESULTS**			
Study selection	16a	Describe the results of the search and selection process, from the number of records identified in the search to the number of studies included in the review, ideally using a flow diagram.	Figure 1 Flow chart
	16b	Cite studies that might appear to meet the inclusion criteria, but which were excluded, and explain why they were excluded.	Figure 1 Flow chart
Study characteristics	17	Cite each included study and present its characteristics.	Table 1, Table 2, Table 4, Table 5, Table 6
Risk of bias in studies	18	Present assessments of risk of bias for each included study.	ND except meta-analysis of sub-cohorts
Results of individual studies	19	For all outcomes, present, for each study: (a) summary statistics for each group (where appropriate) and (b) an effect estimates and its precision (e.g. confidence/credible interval), ideally using structured tables or plots.	Table 2
Results of syntheses	20a	For each synthesis, briefly summarise the characteristics and risk of bias among contributing studies.	Table 1, Table 2, Figure 4, Supplementary material S3, S4, S5
	20b	Present results of all statistical syntheses conducted. If meta-analysis was done, present for each the summary estimate and its precision (e.g. confidence/credible interval) and measures of statistical heterogeneity. If comparing groups, describe the direction of the effect.	&3.1 &3.3
	20c	Present results of all investigations of possible causes of heterogeneity among study results.	&3.2 &4.4
	20d	Present results of all sensitivity analyses conducted to assess the robustness of the synthesized results.	Figure 3
Reporting biases	21	Present assessments of risk of bias due to missing results (arising from reporting biases) for each synthesis assessed.	&4.3 &4.4 &4.5
Certainty of evidence	22	Present assessments of certainty (or confidence) in the body of evidence for each outcome assessed.	&4.3 &4.4 &4.5 &4.6
**DISCUSSION**			
Discussion	23a	Provide a general interpretation of the results in the context of other evidence.	&4.2 &4.3 &4.4 &4.5. &4.6
	23b	Discuss any limitations of the evidence included in the review.	&4.7

	23c	Discuss any limitations of the review processes used.	&4.7
	23d	Discuss implications of the results for practice, policy, and future research.	&4.2 &4.3 &4.4 &4.5 &4.6
**OTHER INFORMATION**			
Registration and protocol	24a	Provide registration information for the review, including register name and registration number, or state that the review was not registered.	Prospero CRD42023396960
	24b	Indicate where the review protocol can be accessed, or state that a protocol was not prepared.	CF PROSPERO
	24c	Describe and explain any amendments to information provided at registration or in the protocol.	&1 &2.1
Support	25	Describe sources of financial or non-financial support for the review, and the role of the funders or sponsors in the review.	No support
Competing interests	26	Declare any competing interests of review authors.	No conflict of interest
Availability of data, code and other materials	27	Report which of the following are publicly available and where they can be found: template data collection forms; data extracted from included studies; data used for all analyses; analytic code; any other materials used in the review.	Source data are mainly public and available (but no template collection form available)

From: Page MJ, McKenzie JE, Bossuyt PM, Boutron I, Hoffmann TC, Mulrow CD, et al. The PRISMA 2020 statement: an updated guideline for reporting systematic reviews. BMJ 2021;372:n71. doi: 10.1136/bmj.n71

For more information, visit: http://www.prisma-statement.org/

Table S2. Medline Query for initial selection of articles to include in the general review.

**The search was conducted from the period 2010 to the date of the request on the 15/03/2023 using the filter "Human" and three keywords (e-cigarette, cigarette, teenager) and their synonyms:**
((**e-cigarette*[**Title/Abstract]) OR (electronic cigarette*[Title/Abstract]) OR (ENDS[Title/Abstract]) OR (vape*[Title/Abstract]) OR (vaping*[Title/Abstract]))
AND ((**cigarette*[**Title/Abstract]) OR (tobacco [Title/Abstract]) OR (smoking [Title/Abstract]))
AND ((teenager*[Title/Abstract]) OR (young*[Title/Abstract]) OR (youth [Title/Abstract]) OR (adolescent*[Title/Abstract]))
AND ((english [Language]) OR (French [Language])) AND (("2011/01/01"[Date-Publication]: "2023/02/31"[Date - Publication]))

Note. We test some variation of the query, as same information with suppression of the filter ("human"), or use of other synonyms.

Table S3. Scoring of causality score of e-cigarettes experimentation or use at T1 on the cigarette consumption at T2 and scoring *of requests of the authors to change the e-cigarette regulation* to restrict use of e-cigarette in the general population of teenagers or young adults.

Scoring	0	1	2	3
causality score	No	Assumption	Possible	Very likely or certain
score of requests to change the e-cigarette regulation	No or ND	Raise the question	Request evolution	Strong request

Note. 5 authors assess the selection of abstract, discussion and conclusions of original article about link e-cigarette/cigarette in each of the 23 publications and propose on an Excel table to score from 0 to 3 each study with a causal score and a score of requests to change the e-cigarette regulation: 46 scores requested for the first round.

Results are sent to the administrator to compile results and organize the progressive consensus. If a perfect consensus exists between the 5 authors for one item, the score is definitive.

A second round of scoring is sent with the anonymous report of the scores of the previous rounds and a new score is request.

A total of 5 rounds has been necessary to reach a consensus for the *causality score* and of 5 rounds for the *score of requests to change the e-cigarette regulation*. The fifth meeting has been organised with online presence of the 5 authors to solve the 2 last discordances and obtain a consensus.

Table S4. Type of consumption (only experimentation, use or daily use) of e-cigarettes at T1 and of cigarettes at T2.

Authors [ref]	E-cigarette experimentation at T1 (1=yes)	E-cigarette use at T1 (1=yes)	Daily e-cigarette use at T1 (1=yes)	Cigarette experimentation at T2 (1=yes)	Cigarette use at T2 (1=yes)	Daily cigarette use at T2 (1=yes)	Observations T2
Leventhal USA [32]	1			1			
Primack USA [33]	1			1			
Barrington-Trimis 2016 USA [34]		1		1	1		30 days
Wills 2016(2) TAIWAN [35]	1			1			
Best UK [36]	1			1			
Hammond CANADA [37]		1			1	1	
Lozano MEXIQUE [38]	1			1			
Miech USA [39]	1				1		12 months
Spindle USA [40]	1			1			
Wills(1) 2017 USA [41]	1			1			
Aleyan CANADA [42]		1		1			
Barrington-Trimis 2018 USA [43]		1		1	1		
Conner UK [26]	1			1			
East UK [44]	1			1			
Loukas USA [45]	1			1			
Morgenstern GERMANY [46]	1			1			
Berry USA [47]	1			1	1		30 days
Chien TAIWAN [48]	1			1			
Kinnunen FINLAND [49]	1					1	
Sun UK [50]	1			1	1		30days/month
Watkins USA [51]	1					1	
Martinelli NEDERLAND [52]	1				1		6/12 months
Osibogun USA [53]		1			1		30 days/month
	18	5	0	17	8	3	

Table S5. Additional information's on populations included at T1 and assessed for cigarette experimentation or use at T2 used to constitute and analyse the 22 sub-cohorts.

Authors [ref]	Smokers excluded from whole cohort analysis to constitute sub-cohort of non-smoker at T1 (n)	source of data	Remarks
Leventhal USA [32]	768	data from authors	From flow-chart F1 (exclusion of all smoker).
Primack USA [33]	94	from authors and from NYTS base	Data from NYTS (13.6% of cigarette smokers for this population) and authors 690 non-smokers according to authors.
Barrington-Trimis 2016 USA [34]	390	data from authors	From Flow chart of authors;
Wills 2016(2) TAIWAN [35]	133	calculate from author datas	Calculated from rate of smokers on the original cohort (longitudinal sub-cohort is only a part of the cohort)
Best UK [36]	570	calculate from author datas	The puffers were included,
Hammond CANADA [37]	1992	calculate from author datas	By authors, but mistake of author in abstract where included (Nb: 19130 in abstract instead 19310 in table i) (puffer considered as non-smoker)
Lozano MEXIQUE [38]	1311	data from authors	estimation of the rate of smoker among the small group of cocaine and cannabis users (we arbitrary chose the rate of non-cannabis or cocaine users group = 20%).
Miech USA [39]	101	data from authors	Calculation of number of smokers from the rate of smokers in initial cohort.
Spindle USA [40]	833	data from authors	
Wills(1) 2017 USA [41]	351	data from authors	
Aleyan CANADA [42]	1527	data from authors	
Barrington-Trimis 2018 USA [43]	910	data from authors	
Conner UK [26]	318	data from authors	
East UK [44]	229	data from authors	Data from authors.
Loukas USA [45]	2017	estimate from Mpact study available	We use 37% of smoker in Mpact cohort in another study to calculate the number smokers excluded. Doi: 10.5993/AJHB.40.4.13
Morgenstern GERMANY [46]	1141	calculate from author datas	With assumption that the rate of the main in loss of follow-up group than in the population analysed (15.5% loss of follow up).
Berry USA [47]	1270	data from authors	
Chien TAIWAN [48]	2841	data from authors	
Kinnunen FINLAND [49]	1309	data from authors	Nb. lost of follow up is not properly describe (37.40%).
Sun UK [50]	1071	estimate from PATH study available	All number provide by authors excepted smoker excluded estimate 12.4% on other PATH cohort same age and same year. https://www.icpsr.umich.edu/files/NAHDAP/pathstudy/Youth-Ever-Cigarette.pdf;
Watkins USA [51]	1612	data from authors	cigarette and other tobacco products are not separate.
Martinelli NEDERLAND [52]	609	data from authors	
Osibogun USA [53]	1837	estimate from PATH study available	Estimation of smoker excluded on PATH data for age and period of survey. Addiction of T2 user for the cohort 6 and the cohort 12 months. https://www.icpsr.umich.edu/files/NAHDAP/pathstudy/Youth-Ever-Cigarette.pdf

Table S6. Sources of the figures used to recalculate the number of smokers initially excluded in the 22 cohorts.

Authors [Ref]	Smokers excluded from analysis of whole cohort to constitute sub-cohort of non-smoker at T1 (n)	T2 cigarette initiation in e-cigarette user T1 group (n)	T2 cigarette initiation in naïve T1 group (n)	T2 initiation of cigarette smoking in sub-cohort	Ratio of T2 cigarette smokers in the reconstituted cohort coming for the group of only e-cigarette users at T1	Ratio of T2 cigarette smoker in the reconstituted cohort coming for the group of naive e-cigarette and cigarette young at T1	Ratio of T2 smoker excluded from sub-cohort before T1 (reintegration for this analysis of T2 at least experimenters of cigarette)
Leventhal USA [32]	768	56	128	184	5.39%	20.69%	73.92%
Primack USA [33]	94	11	128	139	1.20%	15.11%	83.70%
Barrington-Trimis 2016 USA [34]	390	59	111	170	29.06%	36.95%	33.99%
Wills 2016(2) TAIWAN [35]	133	42	50	92	13.95%	16.61%	69.44%
Best UK [36]	571	74	249	323	7.77%	33.93%	58.30%
Hammond CANADA [37]	1992	33	1313	1346	3.80%	40.51%	55.69%
Lozano MEXIQUE [38]	1311	101	1070	1171	3.43%	37.17%	59.40%
Miech USA [39]	101	3	16	19	3.20%	16.00%	80.80%
Spindle USA [40]	833	37	154	191	2.71%	16.56%	80.73%
Wills(1) 2017 USA [41]	351	41	50	91	16.72%	16.08%	67.20%
Aleyan CANADA [42]	1527	112	263	375	3.31%	53.10%	43.59%
Barrington-Trimis 2018 USA [43]	910	184	280	464	13.39%	20.38%	66.23%
Conner UK [26]	318	100	124	224	14.07%	13.28%	72.64%
East UK [44]	229	11	74	85	3.53%	23.08%	73.40%
Loukas USA [45]	2017	114	168	282	4.94%	7.27%	87.79%
Morgenstern GERMANY [46]	1141	72	196	268	3.27%	12.51%	84.23%
Berry USA [47]	1270	108	173	281	6.79%	13.39%	79.82%
Chien TAIWAN [48]	2841	118	1115	1233	2.99%	25.27%	71.74%
Kinnunen FINLAND [49]	1309	9	15	24	3.81%	6.36%	89.83%
Sun UK [50]	1071	221	195	416	6.31%	9.70%	83.98%
Watkins USA [51]	1612	81	387	468	3.89%	18.61%	77.50%
Martinelli NEDERLAND [52]	609	72	128	200	5.51%	16.41%	78.08%
Osibogun USA [53]	1837	17	79	96	1.6%	7.2%	91.2%
mean	1010	73	281	354	*5.34%*	*20.61%*	*74.05%*
total	23235	1676	6466	8142			

Table S7. Detail of progressive consensus of 5 experts with 5 successive votes to reach an agreement on the score of authors conclusions on the causality (*Gateway effect* of e-cigarette on cigarette initiation in adolescents) and on the proposal to reinforce regulation and to decreasing attractivity of the e-cigarette.

	Cotation of causality (gateway effet of e-cigarette on cigarette initiatio																								
Authors [references]	V1 causal					V2 causal					V3 causal					V4 causal					V5 causal				
Leventhal USA [32]	2	2	2	3	2	2	2	2	3	2	2	2	2	2	2	2	2	2	2	2	2	2	2	2	2
Primack USA [33]	2	2	3	3	2	2	3	2	3	1	2	3	2	3	3	3	3	3	3	3	3	3	3	3	3
Barrington-Trimis 2016 USA [34]	2	2	2	2	2	2	2	2	2	2	2	2	2	2	2	2	2	2	2	2	2	2	2	2	2
Wills 2016(2) TAIWAN [35]	2	2	3	3	1	2	3	2	3	3	3	2	3	3	3	3	3	3	3	3	3	3	3	3	3
Best UK [36]	0	1	3	2	0	1	2	0	2	0	2	2	2	2	2	2	2	2	2	2	2	2	2	2	2
Hammond CANADA [37]	1	2	3	3	1	2	2	2	3	2	2	2	2	2	2	2	2	2	2	2	2	2	2	2	2
Lozano MEXIQUE [38]	1	2	2	3	1	1	2	2	2	2	2	2	2	2	2	2	2	2	2	2	2	2	2	2	2
Miech USA [39]	3	3	3	3	3	3	3	3	3	3	3	3	3	3	3	3	3	3	3	3	3	3	3	3	3
Spindle USA [40]	1	2	3	3	0	2	2	2	2	2	2	2	2	2	2	2	2	2	2	2	2	2	2	2	2
Wills(1) 2017 USA [41]	2	2	3	3	1	2	2	2	3	2	2	2	2	2	2	2	2	2	2	2	2	2	2	2	2
Aleyan CANADA [42]	2	2	3	3	2	2	2	2	3	2	2	2	2	2	2	2	2	2	2	2	2	2	2	2	2
Barrington-Trimis 2018 USA [43]	2	2	3	3	3	2	3	3	3	2	3	3	3	3	3	3	3	3	3	3	3	3	3	3	3
Conner UK [26]	2	1	1	0	2	1	1	1	1	2	1	1	1	1	1	1	1	1	1	1	1	1	1	1	1
East UK [44]	1	2	2	1	2	2	2	2	2	2	2	2	2	2	2	2	2	2	2	2	2	2	2	2	2
Loukas USA [45]	1	0	2	3	0	1	0	2	2	2	2	2	2	2	2	2	2	2	2	2	2	2	2	2	2
Morgenstern GERMANY [46]	1	1	2	2	2	2	2	2	2	1	0	2	3	2	2	2	2	2	2	2	2	2	2	2	2
Berry USA [47]	3	3	3	3	3	3	3	3	3	3	3	3	3	3	3	3	3	3	3	3	3	3	3	3	3
Chien TAIWAN [48]	3	3	3	3	3	3	3	3	3	3	3	3	3	3	3	3	3	3	3	3	3	3	3	3	3
Kinnunen FINLAND [49]	3	3	3	2	2	3	3	3	2	3	3	3	3	3	3	3	3	3	3	3	3	3	3	3	3
Sun UK [50]	2	1	2	2	1	2	1	2	2	2	2	2	2	2	2	2	2	2	2	2	2	2	2	2	2
Watkins USA [51]	3	3	3	3	2	3	3	3	3	3	3	3	3	3	3	3	3	3	3	3	3	3	3	3	3
Martinelli NEDERLAND [52]	0	0	0	0	0	0	0	0	0	0	0	0	0	0	0	0	0	0	0	0	0	0	0	0	0
Osibogun USA [53]	3	2	2	2	3	2	2	2	2	3	2	2	2	2	2	2	2	2	2	2	2	2	2	2	2

	Cotation of quiery to change to règles to decrease e-cigarette use (0-																									Final	
Authors [references]	V1 ch.rules					V2 ch.rules					V3 ch.rules					V4 ch.rules					V5 ch.rules					causal	ch.rules
Leventhal USA [32]	0	0	0	0	0	0	0	0	0	0	0	0	0	0	0	0	0	0	0	0	0	0	0	0	0	2	0
Primack USA [33]	3	3	2	1	3	3	3	2	2	2	2	3	2	2	2	2	2	2	2	2	2	2	2	2	2	3	2
Barrington-Trimis 2016 USA [34]	0	1	0	0	2	0	0	0	0	2	0	0	0	0	0	0	0	0	0	0	0	0	0	0	0	2	0
Wills 2016(2) TAIWAN [35]	2	2	2	3	3	2	2	2	3	2	2	2	2	2	2	2	2	2	2	2	2	2	2	2	2	3	2
Best UK [36]	0	0	0	0	1	0	0	0	0	1	0	0	0	0	0	0	0	0	0	0	0	0	0	0	0	2	0
Hammond CANADA [37]	2	2	2	2	2	2	2	2	2	2	2	2	2	2	2	2	2	2	2	2	2	2	2	2	2	2	2
Lozano MEXIQUE [38]	1	2	1	2	3	2	1	2	2	1	2	2	2	2	2	2	2	2	2	2	2	2	2	2	2	2	2
Miech USA [39]	3	2	3	3	3	3	3	3	3	2	3	3	3	3	3	3	3	3	3	3	3	3	3	3	3	3	3
Spindle USA [40]	2	2	2	2	2	2	2	2	2	2	2	2	2	2	2	2	2	2	2	2	2	2	2	2	2	2	2
Wills(1) 2017 USA [41]	2	2	3	3	3	2	2	3	3	3	2	2	3	3	3	3	3	3	3	3	3	3	3	3	3	2	3
Aleyan CANADA [42]	2	3	2	3	3	2	2	3	3	2	2	2	3	3	3	2	3	3	3	3	3	3	3	3	3	2	3
Barrington-Trimis 2018 USA [43]	1	3	2	3	3	2	2	3	3	3	2	3	3	3	3	2	3	3	3	3	3	3	3	3	3	3	3
Conner UK [26]	0	1	0	0	1	0	0	0	0	0	0	0	0	0	0	0	0	0	0	0	0	0	0	0	0	1	0
East UK [44]	2	1	1	1	2	1	2	1	1	1	1	1	1	1	1	1	1	1	1	1	1	1	1	1	1	2	1
Loukas USA [45]	3	3	3	3	3	3	3	3	3	3	3	3	3	3	3	3	3	3	3	3	3	3	3	3	3	2	3
Morgenstern GERMANY [46]	0	0	2	1	0	0	0	0	0	1	0	0	3	0	0	0	0	0	0	0	0	0	0	0	0	2	0
Berry USA [47]	3	3	3	3	3	3	3	3	3	3	3	3	3	3	3	3	3	3	3	3	3	3	3	3	3	3	3
Chien TAIWAN [48]	3	3	3	3	3	3	3	3	3	3	3	3	3	3	3	3	3	3	3	3	3	3	3	3	3	3	3
Kinnunen FINLAND [49]	2	2	2	2	2	2	2	2	2	2	2	2	2	2	2	2	2	2	2	2	2	2	2	2	2	3	2
Sun UK [50]	0	0	0	0	0	0	0	0	0	0	0	0	0	0	0	0	0	0	0	0	0	0	0	0	0	2	0
Watkins USA [51]	2	2	3	2	3	2	2	2	2	2	2	2	2	2	2	2	2	2	2	2	2	2	2	2	2	3	2
Martinelli NEDERLAND [52]	0	0	0	0	0	0	0	0	0	0	0	0	0	0	0	0	0	0	0	0	0	0	0	0	0	0	0
Osibogun USA [53]	2	3	1	2	2	2	2	2	2	2	2	2	2	2	2	2	2	2	2	2	2	2	2	2	2	2	2

Table S8. Influence of first product experimented (cigarette or e-cigarette) on the subsequent consumption of the other.

Authors [Ref]	Transition from experimentation e-cigarette at T1 to experimentation cigarette at T2 ORa (95%CI)	Transition from experimentation e-cigarette at T1 to cigarette use at T2 ORa (95%CI)	Causal mediation analysis from e-cigarette experimentation at T1 to at T2 cigarette use (OR 95%CI)	Transition from experimentation cigarette at T1 to experimentation e-cigarette at T2 ORa (95% CI)	Transition from experimentation cigarette at T1 to e-cigarette use at T2 ORa (95% CI)	Causal mediation analysis from cigarette experimentation at T1 to at T2 e-cigarette use OR (95% CI)
Barrington-Trimis et al. [37] *	4.58 (3.56-5.88)	3.51 (1.97-6.24)		4.03 (1.30-12.60)	28.8 (12.6-66.1)	
Martinelli et al. [46] *	4.58 (2.42-8.68)			3.52 (2.02. 6.11)		
East et al. [38] *	3.54 (1.68-7.45)	5.79 (2.55-13.15)	1.34 (1.05-1.72)	11.89 (3.56-39.72)	7.89 (3.06-20.38)	1.08 (1.01-1.17)
Penzes et al. [53]	3.57 (1.96-6.49)			3.78 (2.66-5.37)		
Bold et al. [54]	7.08 (2.34-21.42)			2.02 (0.67-6.08)		
	3.87 (1.86-8.06)			1.90 (0.77-4.71)		
Kang et al. [22]		6.8 (4.5-10.2)			44.1 (34.1-56.9)	
Staff et al. [55]	5.09 (4.01-6.47)	5.05 (3.82-6.69)		2.11 (1.46-3.06)	2.89 (1.64-5.10)	
Aleyan et al. [56]		1.54 (1.37-1.74)			1.43 (1.33-1.58)	
		1.18 (1.08-1.29)			1.07 (0.99-1.15)	
	4.62	4.18		5.37	7.00	

Note: * include in the sub-cohort analyzised
